# TDAG51 deficiency attenuates dextran sulfate sodium-induced colitis in mice

**DOI:** 10.1038/s41598-022-24873-4

**Published:** 2022-11-30

**Authors:** Hyoeun Jeon, Dulshara Sachini Amarasekara, Nari Lee, Hye-Won Park, Jiyeon Yu, Jaerang Rho

**Affiliations:** Department of Microbiology and Molecular Biology, College of Bioscience and Biotechnology, Chungnam National University, 99 Daehak-ro, Yuseong-gu, Daejeon, 34134 Korea

**Keywords:** Genetics, Immunology, Molecular biology, Gastroenterology, Molecular medicine

## Abstract

Inflammatory bowel disease (IBD), including ulcerative colitis and Crohn’s disease, is a group of chronic inflammatory diseases of the gastrointestinal tract. Although the multifactorial etiology of IBD pathogenesis is relatively well documented, the regulatory factors that confer a risk of IBD pathogenesis remain less explored. In this study, we report that T-cell death-associated gene 51 (TDAG51/PHLDA1) is a novel regulator of the development of dextran sulfate sodium (DSS)-induced colitis in mice. TDAG51 expression was elevated in the colon tissues of DSS-induced experimental colitis mice. TDAG51 deficiency protected mice against acute DSS-induced lethality and body weight changes and disease severity. DSS-induced structural damage and mucus secretion in colon tissues were significantly reduced in TDAG51-deficient mice compared with wild-type mice. We observed similar results in a DSS-induced chronic colitis mouse model. Finally, we showed that the production of inflammatory mediators, including proinflammatory enzymes, molecules and cytokines, was decreased in DSS-treated TDAG51-deficient mice compared with DSS-treated wild-type mice. Thus, we demonstrated that TDAG51 deficiency plays a protective role against DSS-induced colitis by decreasing the production of inflammatory mediators in mice. These findings suggest that TDAG51 is a novel regulator of the development of DSS-induced colitis and is a potential therapeutic target for IBD.

## Introduction

Inflammatory bowel disease (IBD) refers to a group of chronic inflammatory conditions of the gastrointestinal tract (GIT), of which Crohn’s disease (CD) and ulcerative colitis (UC) have the highest global prevalence^1^. Both CD and UC patients exhibit recurrent diarrhea, bloody stools, fatigue and weight loss^1^. UC is characterized by prolonged chronic inflammation of the colonic mucosa, whereas inflammation manifests throughout the GIT in CD^2^. However, the key genetic or environmental risk factors for IBD remain elusive^3^. Chronic inflammation caused by dysregulated immune function in the colonic mucosa due to environmental triggers in genetically susceptible individuals could be the best possible explanation for the etiology of IBD^4^.

The pathogenesis of IBD is an intricate process that involves epithelial cell damage, excessive immune cell activation and aberrant cytokine response^5,6^. In particular, proinflammatory cytokines, such as interleukin (IL)-1β, IL-6 and tumor necrosis factor (TNF)-α, play vital roles in IBD pathogenesis, disease progression and disease resolution^7^. Elevated levels of TNF-α are closely associated with IBD severity and provoke an inflammatory response in the GIT by inducing IL-1β and IL-6 production through nuclear factor-κB (NF-κB) activation^6,7^. Massive release of proinflammatory cytokines by activated immune cells in response to a trigger of IBD flares in the gut mucosa exacerbates the dysfunctional immune response of innate immune cells and even CD4^+^ Th1, Th2 and Th17 cells, thereby leading to damage to the intestinal epithelial barrier associated with chronic inflammation^5,8^. Although the multifactorial etiology of IBD pathogenesis is relatively well documented, the regulatory factors that confer a risk of IBD pathogenesis remain less explored.

T-cell death-associated gene 51 (TDAG51), also known as pleckstrin homology-like (PHL) domain family A1 (PHLDA1), is vitally involved in regulating apoptosis^9–12^. TDAG51 is ubiquitously expressed in most organs, such as the brain, liver, lung, intestine, heart, kidney, thymus, lymph nodes and spleen^10,11,13^. In particular, TDAG51 is markedly expressed in human intestinal tissues and even in intestinal tumors^14^. TDAG51 is a marker of putative epithelial stem cells in the small and large intestines^15^. Furthermore, recent studies have reported that TDAG51 expression is elevated in human UC and UC-associated colorectal cancer, indicating that TDAG51 expression is linked to the pathogenesis of UC^16,17^. TDAG51 expression is induced by various cellular stress responses, such as heat shock, endoplasmic reticulum stress and oxidative stress^9,10,12,18,19^. TDAG51 expression is also induced by pathogen infection and inflammation^20–23^. Interestingly, it has been reported that TDAG51 expression is rapidly induced by the Toll-like receptor (TLR) 2/4-mediated signaling pathway^20,21,23,24^. Moreover, TDAG51 deficiency exerts a protective effect against acute- or chronic inflammation-associated disorders, such as atherosclerosis, obesity, neuroinflammation, ischemia/reperfusion injury and lung injury^13,18,20,23–26^. Thus, we hypothesized that the functional role of TDAG51 is possibly linked to the regulation of the inflammatory response in intestinal tissues.

The dextran sulfate sodium (DSS)-induced colitis mouse model is a relatively well-studied experimental animal model of IBD^27,28^. In mice, acute colitis is commonly induced by continuous oral administration of 2–5% DSS for a short period (4–9 days), while chronic colitis is usually induced by cyclical oral administration of low concentrations of DSS^27–29^. The symptoms of DSS-induced experimental colitis model mice mimic the symptoms of human acute and chronic UC, including weight loss, bloody diarrhea, shortening of the colon and epithelial barrier damage, with elevated levels of proinflammatory cytokines in the intestinal mucosa^27–29^.

In this study, we demonstrated that TDAG51 is a novel regulator in a DSS-induced experimental colitis mouse model. TDAG51-deficient (TDAG51^−/−^) mice with DSS-induced colitis showed reduced disease severity and increased survival. TDAG51 deficiency attenuated the production of inflammatory mediators. Thus, we report that TDAG51 deficiency protects against DSS-induced colitis by decreasing the production of inflammatory mediators.

## Results

### TDAG51 expression in mouse colon tissues is elevated by oral DSS administration

**Figure 1:**
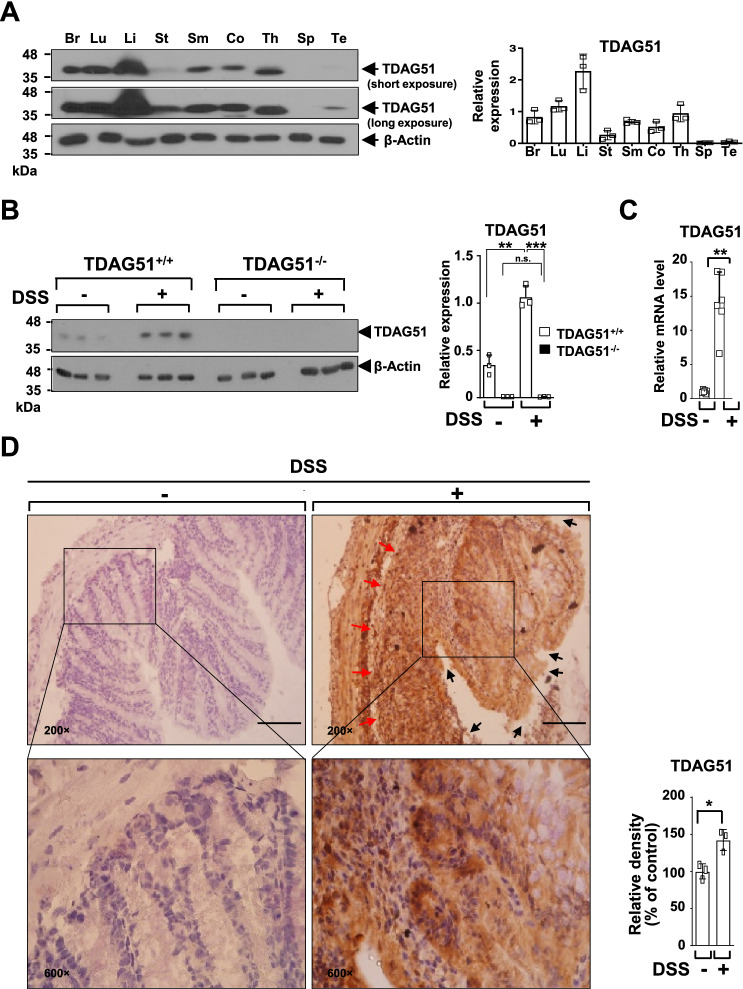
TDAG51 expression is elevated in the colon tissues of DSS-induced experimental colitis model mice. (**A**) TDAG51 expression in various mouse tissues. Organs and tissues were collected from 8-week-old C57BL/6J male mice. β-Actin was used as a loading control. The relative levels of TDAG51 from immunoblotting with an anti-TDAG51 antibody (left panel) were quantified by densitometry and normalized to β-actin (right panel). *Br* brain, *Lu* lung, *Li* liver, *St* stomach, *Sm* small intestine, *Co* colon, *Th* thymus, *Sp* spleen, *Te* testis. (**B**) Elevated TDAG51 expression in the colon tissues of DSS-induced experimental colitis model mice. C57BL/6J male mice (8 weeks old, n = 12 per group) were administered 1.7% DSS in drinking water for 4 days followed by water for 3 days in 3 cycles. Three randomly selected mice in each group were sacrificed on day 21. The protein levels of TDAG51 in the distal anorectal region of colon tissues were analyzed by immunoblotting (left panel) and quantification (right panel). (**C**) The relative mRNA levels of TDAG51. The distal anorectal region of colon tissues of DSS-induced colitis mice was subjected to real-time PCR analysis. The data were normalized to β-actin levels. (**D**) TDAG51 expression levels in the colon tissues of DSS-induced colitis model mice. The distal anorectal region of colon tissues of DSS-induced colitis mice was sectioned and analyzed by immunohistochemistry with an anti-TDAG51 antibody and hematoxylin (left panel; original magnification, ×200  and ×600). The black arrow marks destruction of structural integrity; the red arrow indicates infiltration of immune cells. The relative expression of TDAG51 was analyzed (right panel) by comparison of immunopositive cells (brown) with relevant counterstained cells (purple nuclei). Scale bars, 100 μm. ^*^P < 0.05. ^**^P < 0.01. ^***^P < 0.001.

To analyze the role of TDAG51 in an experimental colitis mouse model, we first examined the expression levels of TDAG51 in mouse colon tissues. TDAG51 was detected in mouse colon tissues, but TDAG51 expression levels were relatively lower in the colon than in the brain, liver, lung and thymus (Fig. 1A). We next analyzed whether DSS-induced colitis induces TDAG51 expression in mouse colon tissues. To evaluate the induction of TDAG51 expression, the distal anorectal region of colon tissues was collected from DSS-induced chronic colitis model mice at day 21 and subjected to immunoblotting analysis with an anti-TDAG51 antibody. We observed that TDAG51 expression was elevated in the distal anorectal region of colon tissues of DSS-induced colitis model mice compared to those of control mice, whereas the basal level and DSS-induced induction of TDAG51 expression were not detected in the distal anorectal region of colon tissues in TDAG51^−/−^ mice (Fig. 1B). Real-time PCR analysis revealed that the mRNA levels of TDAG51 were significantly increased in the distal anorectal region of colon tissues of DSS-induced colitis mice (Fig. 1C). Consistent with these results, immunohistochemistry showed that the number of cells stained with an anti-TDAG51 antibody was increased in the distal anorectal region of colon tissues of DSS-induced colitis mice compared to those of control mice (Fig. 1D). These results indicate that TDAG51 expression is elevated in the distal anorectal region of colon tissues of DSS-induced colitis model mice.

### TDAG51 deficiency protects mice from DSS-induced acute colitis

**Figure 2:**
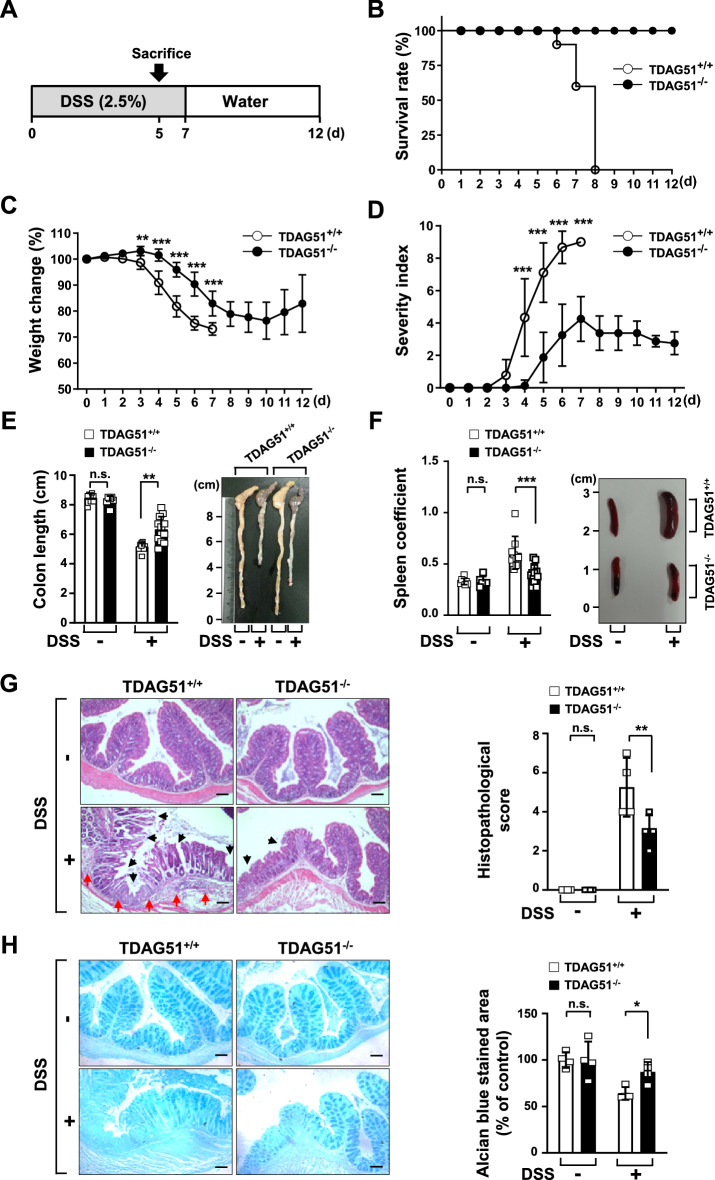
TDAG51 deficiency attenuates DSS-induced acute colitis. (**A**) Schematic diagram of DSS-induced acute colitis. C57BL/6J male mice (8 weeks old, n = 8–9 per group) were administered 2.5% DSS in drinking water for 7 days followed by water for 5 days. (**B**) DSS-induced lethality. The survival rates of DSS-treated TDAG51^+/+^ and TDAG51^−/−^ mice were monitored daily. (**C**) Body weight change after DSS administration. The initial body weight of each mouse was defined as 100%. (**D**) The clinical progression of DSS-induced colitis was evaluated by the disease severity index. The disease severity index was calculated as the sum of the severity scores for body weight loss (0–3), stool consistency (0–3) and rectal bleeding (0–3) as described in the “Materials and methods”. (**E**) Comparison of colon length and representative images of colons from control and DSS-induced colitis model mice. The mice (8 weeks old, n = 8–13 per group) were sacrificed on day 5. (**F**) Comparison of spleen coefficients and representative images of spleens from control and DSS-induced colitis model mice. (**G**) Histopathological changes in the distal anorectal region of colon tissues. Colon sections were stained with H&E (left panel; original magnification, ×100). The black arrow marks destruction of structural integrity; the red arrow indicates infiltration of immune cells. Histopathological scores were analyzed (right panel). (**H**) Analysis of mucus secretion in colon tissues. Mucus secretions were analyzed by Alcian blue staining (left panel; original magnification, ×100). The relative blue-stained area was analyzed (right panel). Scale bars, 100 μm. ^*^P < 0.05. ^**^P < 0.01. ^***^P < 0.001. *n.s.* not significant.

DSS-induced acute colitis in TDAG51^−/−^ and TDAG51^+/+^ mice was induced by oral administration of 2.5% DSS in drinking water for 7 consecutive days followed by drinking water for 5 days (Fig. 2A). Mortality and parameters of disease severity in mice with DSS-induced acute colitis were monitored daily. All experimental mice survived 5 days of oral administration of 2.5% DSS (Fig. 2B). The survival rate of TDAG51^+/+^ mice rapidly decreased from day 6 to day 8, and no surviving mice were observed at day 8; however, all TDAG51^−/−^ mice survived (Fig. 2B). Consistent with these results, as shown in Fig. 2B, the body weight of TDAG51^+/+^ mice was markedly decreased after 3 days of oral administration of DSS, whereas the body weight of TDAG51^−/−^ mice was slightly diminished from day 5 to day 10 and partially recovered after day 10 (Fig. 2C). We next analyzed the severity index of DSS-induced acute colitis by calculating overall severity scores for body weight loss (0–3), stool consistency (0–3) and rectal bleeding (0–3). The initial symptoms of DSS-induced acute colitis in TDAG51^+/+^ mice were observed at day 3, and the severity scores were markedly increased until all TDAG51^+/+^ mice died (Fig. 2D). However, TDAG51^−/−^ mice showed initial symptoms at day 5, peaked severity score at day 8 and showed a slight decline in severity after day 8 (Fig. 2D). Compared with TDAG51^−/−^ mice, TDAG51^+/+^ mice showed a 4.8-fold increase in the peak severity of DSS-induced acute colitis (Fig. 2D). Shortening of the colon and spleen enlargement are characteristic features of DSS-induced acute colitis^27,28^. Thus, we further compared the colon length and spleen coefficient. We observed that the colon length of DSS-treated TDAG51^+/+^ mice (5.16 ± 0.28 cm) was shorter than that of DSS-treated TDAG51^−/−^ mice (6.32 ± 0.80 cm), while colon length was not significantly different between the control groups that did not receive DSS (8.03 ± 0.31 or 8.07 ± 0.19 cm) (Fig. 2E). The spleen coefficient was markedly increased in DSS-treated TDAG51^+/+^ mice (0.61 ± 0.15) compared to that in DSS-treated TDAG51^−/−^ mice (0.4 ± 0.09) and control mice not administered DSS (0.33 ± 0.03 or 0.33 ± 0.05) (Fig. 2F). Finally, we compared morphological damage by hematoxylin and eosin (H&E) staining and protective mucus secretion by Alcian blue staining in the distal anorectal region of colon tissues of DSS-treated TDAG51^+/+^ and TDAG51^−/−^ mice. As shown in Fig. 2B–F, DSS-induced destruction of structural integrity, including the epithelial monolayer, villi and crypt structure, and infiltration of immune cells, such as neutrophils, eosinophils, macrophages and lymphocytes, were significantly alleviated in the distal anorectal region of colon tissues of TDAG51^−/−^ mice compared to those of DSS-treated TDAG51^+/+^ mice (Fig. 2G). Furthermore, we observed that protective mucus secretion was restored in the distal anorectal region of colon tissues of DSS-treated TDAG51^−/−^ mice, while DSS-treated TDAG51^+/+^ mice showed reduced mucus secretion (Fig. 2H). However, there were no abnormal histopathological differences in other organs and tissues (for instance, duodenum, jejunum, ileum, stomach, lung, liver and kidney) observed between DSS-treated TDAG51^+/+^ and TDAG51^−/−^ mice (Supplementary Fig. S1). Collectively, these results indicate that TDAG51 deficiency attenuates DSS-induced acute colitis in mice.

### TDAG51 deficiency protects mice from DSS-induced chronic colitis

**Figure 3:**
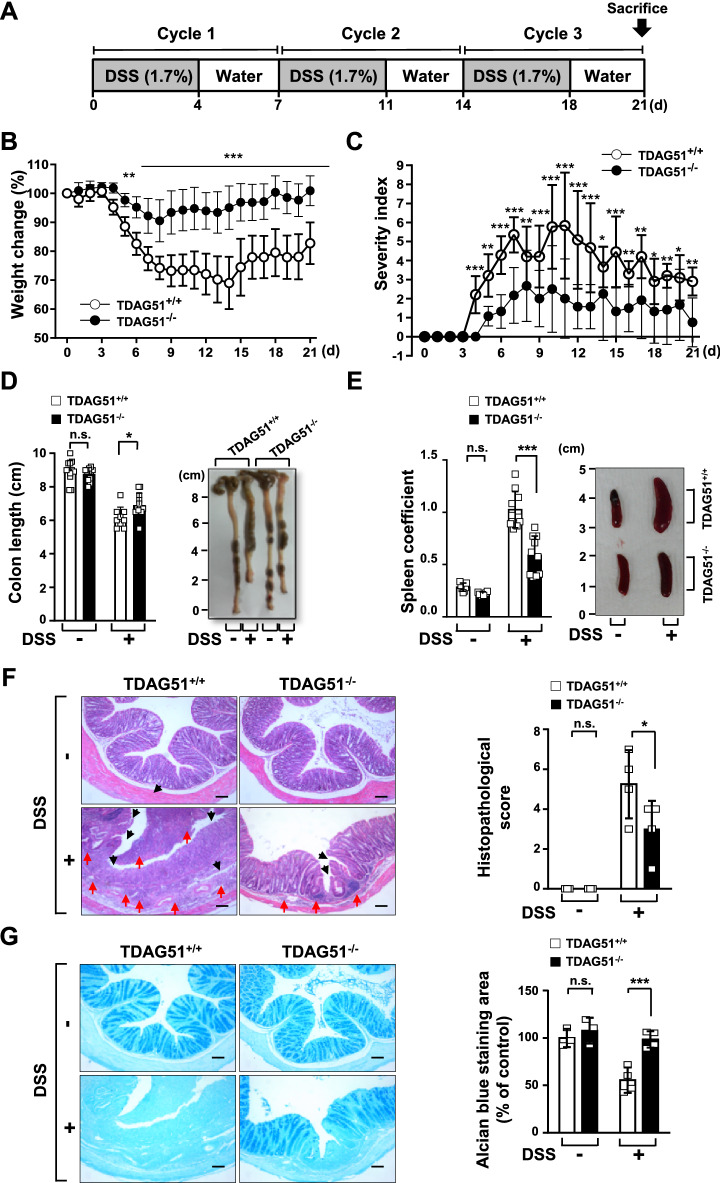
TDAG51 deficiency attenuates DSS-induced chronic colitis. (**A**) Schematic diagram of DSS-induced chronic colitis. C57BL/6 J male mice (8 weeks old, n = 12–14 per group) were administered 1.7% DSS in drinking water for 4 days followed by water for 3 days in 3 cycles. (**B**) Body weight change after DSS administration. The initial body weight of each mouse was defined as 100%. (**C**) The clinical progression of DSS-induced colitis was evaluated by the disease severity index. The disease severity index was calculated as the sum of the severity scores for body weight loss (0–3), stool consistency (0–3) and rectal bleeding (0–3) as described in the “Materials and methods”. (**D**) Comparison of colon length and representative images of colons of controls and DSS-induced colitis mice. (**E**) Comparison of spleen coefficients and representative images of spleens of control and DSS-induced colitis mice. (**F**) Histopathological changes in the distal anorectal region of colon tissues. Colon sections were stained with H&E (left panel; original magnification, ×100). The black arrow marks destruction of structural integrity; the red arrow indicates infiltration of immune cells. Histopathological scores were analyzed (right panel). (**G**) Analysis of mucus secretions in colon tissues. Mucus secretions were analyzed by Alcian blue staining (left panel; original magnification, ×100). The relative blue-stained area was analyzed (right panel). Scale bars, 100 μm. ^*^P < 0.05. ^**^P < 0.01. ^***^P < 0.001. *n.s.* not significant.

DSS-induced chronic colitis was induced in TDAG51^−/−^ and TDAG51^+/+^ mice by 3 cycles of oral administration of 1.7% DSS in drinking water for 4 consecutive days followed by drinking water for 3 days, and disease severity was monitored daily (Fig. 3A). The body weight of TDAG51^+/+^ mice was markedly decreased after 3 days of DSS oral administration in the first cycle and reached the lowest value on day 14, and body weight partially recovered from day 14 to day 21 (Fig. 3B). However, DSS-treated TDAG51^−/−^ mice showed a slight decrease in body weight from day 4 to day 8, and body weight recovered to nearly the baseline level from day 8 to day 21 (Fig. 3B). The severity score was markedly increased in DSS-treated TDAG51^+/+^ mice, while DSS-treated TDAG51^−/−^ mice showed a slight decline in severity (Fig. 3C). Compared to TDAG51^−/−^ mice, TDAG51^+/+^ mice showed a 2.2-fold increase in the peak severity of DSS-induced chronic colitis (Fig. 3C). Similar to the results related to the severity of DSS-induced acute colitis (Fig. 2E,F), the colon length of DSS-treated TDAG51^+/+^ mice (6.17 ± 0.62 cm) was shorter than that of DSS-treated TDAG51^−/−^ mice (6.91 ± 0.70 cm), while there was no significant difference in colon length between the control groups not treated with DSS (8.76 ± 0.35 or 8.93 ± 0.65 cm) (Fig. 3D). The spleen coefficient was markedly increased in DSS-treated TDAG51^+/+^ mice (1.03 ± 0.17) compared to that in DSS-treated TDAG51^−/−^ mice (0.59 ± 0.17) and control mice not treated with DSS (0.28 ± 0.04 or 0.22 ± 0.02) (Fig. 3E). As shown in Fig. 3B–E, DSS-induced destruction of structural integrity and infiltration of immune cells were significantly alleviated in the distal anorectal region of colon tissues of TDAG51^−/−^ mice compared to those of DSS-treated TDAG51^+/+^ mice (Fig. 3F). Furthermore, protective mucus secretion was restored in the distal anorectal region of colon tissues of DSS-treated TDAG51^−/−^ mice, while DSS-treated TDAG51^+/+^ mice showed markedly reduced mucus secretion (Fig. 3G). Collectively, these results indicate that TDAG51 deficiency attenuates DSS-induced chronic colitis in mice.

### TDAG51 deficiency attenuates the production of inflammatory enzymes in DSS-induced colitis model mice

**Figure 4:**
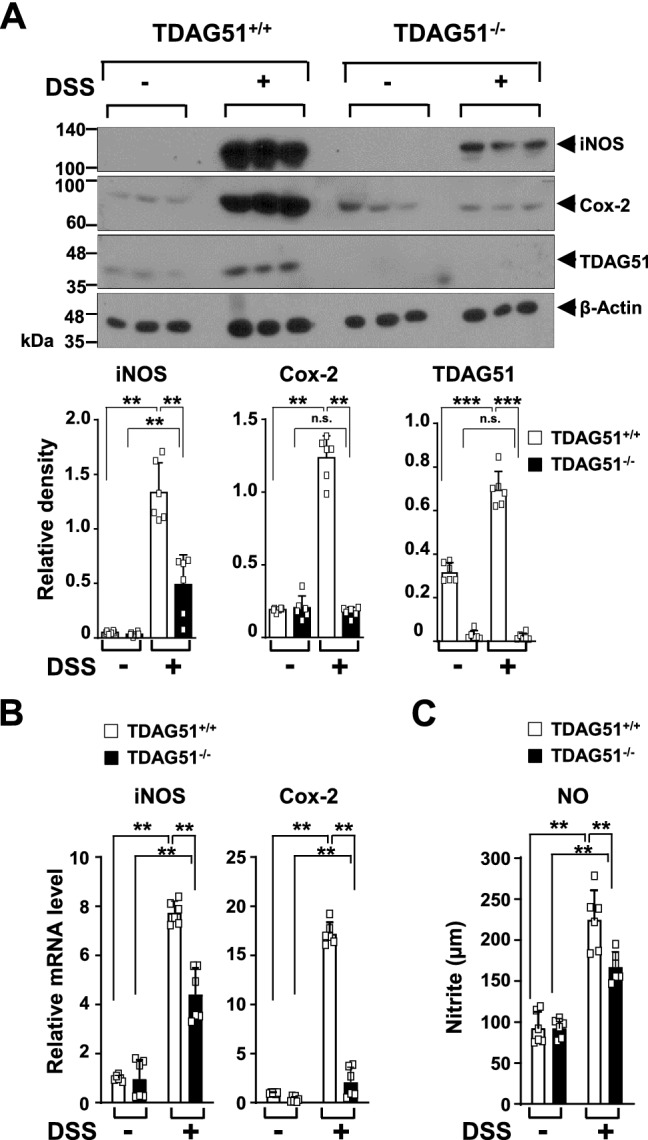
TDAG51 deficiency decreases DSS-induced production of inflammatory enzymes. (**A**) The protein levels of iNOS and Cox-2 were measured by immunoblotting analysis. C57BL/6J male mice (8 weeks old, n = 12–14 per group) were administered 1.7% DSS in drinking water for 4 days followed by water for 3 days in 3 cycles. At day 21, three mice were randomly selected from each group for sacrifice. The distal anorectal region of colons was collected and analyzed by immunoblotting (top panel). The relative density was analyzed (bottom panel). β-Actin was used as a loading control. (**B**) The mRNA levels of iNOS and Cox-2 were measured by real-time PCR. The data were normalized to β-actin levels. (**C**) The production of NO was analyzed. ^**^P < 0.01. ^***^P < 0.001. *n.s.* not significant.

We next analyzed whether TDAG51 expression alters the production of inflammatory enzymes and molecules in the colon tissues of DSS-induced chronic colitis model mice. The protein expression levels of iNOS and Cox-2 were enhanced in the distal anorectal region of colon tissues of DSS-treated mice compared to those of control mice (Fig. 4A). However, the elevation of the protein levels of iNOS and Cox-2 was significantly decreased in the distal anorectal region of colon tissues of DSS-treated TDAG51^−/−^ mice compared to those of DSS-treated TDAG51^+/+^ mice (Fig. 4A). Consistent with these results, compared to DSS-treated TDAG51^+/+^ mice, DSS-treated TDAG51^−/−^ mice showed reduced mRNA levels of iNOS and Cox-2 in the distal anorectal region of colon tissues (Fig. 4B). Furthermore, the production of NO was reduced in the sera of DSS-treated TDAG51^−/−^ mice compared to that in the sera of DSS-treated TDAG51^+/+^ mice (Fig. 4C). Taken together, these results indicate that the induction of TDAG51 expression in the distal anorectal region of colon tissues of mice with DSS-induced colitis is involved in the enhancement of inflammatory enzyme and inflammatory molecule production.

### TDAG51 deficiency attenuates the production of proinflammatory cytokines in DSS-induced colitis model mice

**Figure 5:**
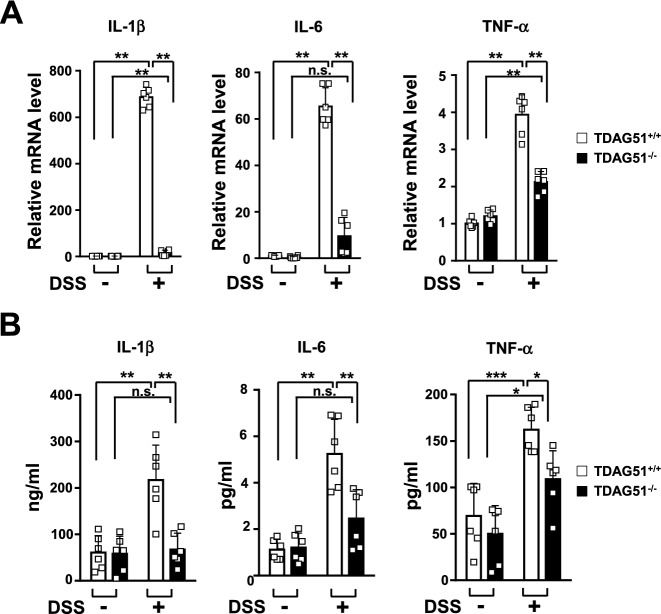
TDAG51 deficiency decreases the DSS-induced production of proinflammatory cytokines. (**A**) The mRNA levels of proinflammatory cytokines were measured by real-time PCR. C57BL/6J male mice (8 weeks old, n = 12–14 per group) were administered 1.7% DSS in drinking water for 4 days followed by water for 3 days in 3 cycles. On day 21, the distal anorectal region of colons was collected and analyzed by real-time PCR. The data were normalized to β-actin levels. (**B**) The serum levels of proinflammatory cytokines were analyzed by ELISA. ^*^P < 0.05. ^**^P < 0.01. ^***^P < 0.001. n.s., not significant.

Elevation of the levels of proinflammatory cytokines is closely associated with DSS-induced colitis in mice and even in patients with IBD^30^. Thus, we examined whether the expression of proinflammatory cytokines is dependent on the availability of TDAG51 in DSS-induced colitis model mice. As shown in Fig. 4, we observed that the mRNA levels of proinflammatory cytokines, including IL-1β, IL-6 and TNF-α, were significantly decreased in the colon tissues of DSS-treated TDAG51^−/−^ mice compared to those of DSS-treated TDAG51^+/+^ mice (Fig. 5A). Similarly, the serum levels of proinflammatory cytokines were reduced in DSS-treated TDAG51^−/−^ mice compared to those in DSS-treated TDAG51^+/+^ mice (Fig. 5B). Taken together, these results indicate that TDAG51 deficiency has a protective role against DSS-induced colitis in mice by decreasing the production of proinflammatory cytokines.

### TDAG51 restores the inhibitory effect of peroxisome proliferator-activated receptor γ (PPARγ) on lipopolysaccharide (LPS)-induced proinflammatory cytokine expression

**Figure 6:**
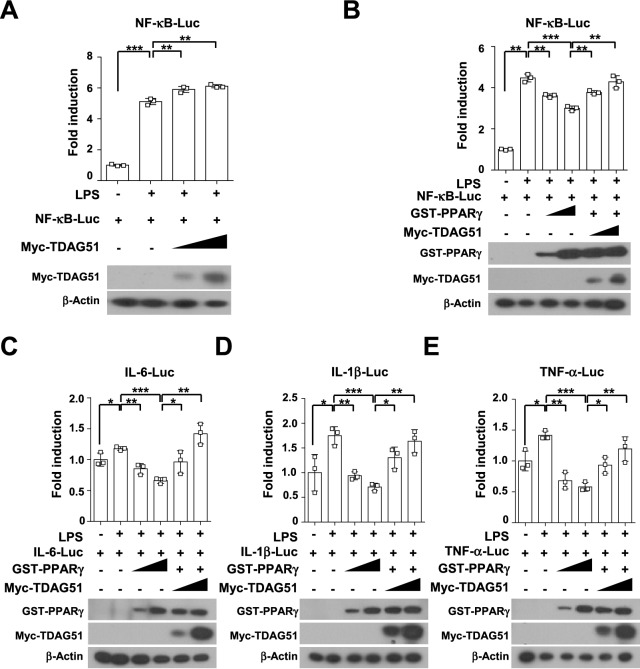
PPARγ-mediated inhibition of LPS-induced NF-κB and proinflammatory cytokine promoter activities is restored by TDAG51 expression. (**A**) LPS-induced NF-κB promoter activity is enhanced by TDAG51 expression. Reporter plasmids [NF-κB-Luc (0.2 μg) and pcDNA3.1/His/LacZ (0.1 μg)] were cotransfected with Myc-tagged TDAG51 expression plasmid [Myc-TDAG51 (0.3–1.0 μg)] into 293/TLR4-MD2-CD14 cells. The transfected cells were stimulated with 1 μg/ml LPS for 24 h and then analyzed using luciferase assays (top panel). The expression of epitope-tagged plasmids was analyzed by immunoblotting against anti-epitope antibodies (bottom panel). β-Actin was used as the loading control. (**B**) PPARγ-mediated inhibition of LPS-induced NF-κB promoter activity is restored by TDAG51 expression. Reporter plasmids [NF-κB-Luc (0.2 μg) and pcDNA3.1/His/LacZ (0.1 μg)] were cotransfected with epitope-tagged expression plasmids [GST-PPARγ (0.25–1.0 μg), Myc-TDAG51 (0.3–1.0 μg)] into 293/TLR4-MD2-CD14 cells. The luciferase activity (top panel) and the expression of epitope-tagged plasmid (bottom panel) were analyzed. (**C**) PPARγ-mediated inhibition of LPS-induced IL-6 promoter activity is restored by TDAG51 expression. (**D**) PPARγ-mediated inhibition of LPS-induced IL-1β promoter activity is restored by TDAG51 expression. (**E**) PPARγ-mediated inhibition of LPS-induced TNF-α promoter activity is restored by TDAG51 expression. ^*^P < 0.05. ^**^P < 0.01. ^***^P < 0.001.

The LPS/TLR-induced signaling pathway is responsible for disease progression by inducing proinflammatory cytokine production in an experimental colitis mouse model^7,31–33^. To investigate whether TDAG51 is involved in LPS-induced proinflammatory cytokine expression, CT26 cells, a murine colorectal carcinoma cell line, were stimulated with LPS (100 ng/ml) for 48 h. Upon LPS stimulation, the level of TDAG51 expression peaked at 2 h and then declined to the basal level until 48 h (Supplementary Fig. S2A). Next, we generated a TDAG51-deficient CT26 clone (CT26^TDAG51−/−^) by introducing the CRISPR‒Cas9 system with guide RNA targeting the PHL domain of TDAG51 in CT26 cells (wild type: CT26^TDAG51+/+^) (Supplementary Fig. S2B). TDAG51 basal expression and induction by LPS stimulation were completely abolished in the CT26^TDAG51−/−^ clone (Supplementary Fig. S2C). However, LPS-induced signaling pathways, including NF-κB and MAP kinase activation, and nuclear translocations of its downstream transcription factors, such as NF-κB and AP-1, were not altered in the CT26^TDAG51−/−^ clone (Supplementary Fig. S2C–F). Thus, these results indicate that the basal level and LPS-induced expression of TDAG51 per se are not involved in the regulation of the LPS-induced signaling pathway. We have recently reported that the interaction of TDAG51 with PPARγ inhibits the transcriptional regulation of PPARγ^34^. The role of PPARγ is negatively linked to the regulation of proinflammatory cytokine expression in IBD pathogenesis^35–38^. Thus, we next analyzed the effects of TDAG51 on LPS-induced promoter activities of proinflammatory cytokine genes using reporter assays. Upon LPS stimulation, the promoter activities of the NF-κB reporter were enhanced by TDAG51 expression in a dose-dependent manner (Fig. 6A), whereas LPS-induced NF-κB reporter activities were dose-dependently inhibited by PPARγ expression (Fig. 6B). The inhibitory effects of PPARγ on LPS-induced NF-κB reporter activities were rescued by TDAG51 expression in a dose-dependent manner (Fig. 6B). Furthermore, the inhibition of PPARγ on LPS-induced promoter activities of proinflammatory cytokine genes was clearly restored by TDAG51 expression in a dose-dependent manner (Fig. 6C–E). Taken together, these results indicate that TDAG51 induction positively regulates LPS-induced proinflammatory cytokine expression by inhibiting the negative role of PPARγ.

## Discussion

TDAG51 is responsible for various physiological and cellular stimuli, such as heat shock, cellular intrinsic stresses, growth factors, infection and inflammation^9,10,12,18–21,34,39^. Our current study reveals that (1) TDAG51 expression is elevated in the distal anorectal region of colon tissues of DSS-induced experimental colitis model mice, (2) TDAG51 deficiency protects mice against DSS-induced lethality and body weight changes and disease severity, and (3) TDAG51 deficiency attenuates the production of inflammatory mediators in a DSS-induced experimental colitis mouse model. Thus, we identified TDAG51 as a novel regulator of DSS-induced colitis in mice.

TDAG51 contains an N-terminal PHL domain, which is mainly involved in protein–protein interactions, C-terminal proline-glutamine repeats and proline-histidine repeats, which are predicted to function as a transactivation domain but lack DNA binding motifs^11,34^. Thus, TDAG51 is considered to be a putative transcriptional regulator involved in diverse biological processes, such as cell fate, the stress response, inflammation, tumor progression and tissue homeostasis^39,40^. Thus, we assume that TDAG51’s function as a transcriptional activator is possibly linked to the regulation of the production of inflammatory mediators in the colon tissues of the DSS-induced experimental colitis model mice. The detrimental effects of proinflammatory cytokines, such as IL-1β, IL-6 and TNF-α, in IBD, particularly in UC, are relatively well documented^5–7^. The expression of proinflammatory cytokines is also closely linked to the induction of inflammatory enzymes, such as iNOS and Cox-2, which are responsible for the production of inflammatory molecules, including NO, reactive oxygen species and prostaglandin E_2,_ to further promote inflammation^41^. Interestingly, it has been reported that the expression levels of Cox-2 and iNOS are elevated in the intestinal tissues of IBD patients and mice with experimental colitis^7,42,43^. Inhibitors of Cox-2 and iNOS suppress disease aggravation in human IBD patients and in mice with experimental colitis^44,45^. In our current study, we clearly showed that TDAG51 deficiency attenuates the production of inflammatory mediators, such as proinflammatory cytokines, enzymes and molecules, in the DSS-induced experimental colitis mouse model (Figs. 4 and 5). Thus, it is possible that via its transactivation function, TDAG51 may elevate the production of inflammatory mediators in the DSS-induced experimental colitis mouse model.

NF-κB activation is responsible for disease progression in human IBD patients and in an experimental colitis mouse model^6,7,46^. Gut microbial antigen sensing mediated by pattern-recognized receptors, such as TLRs, provokes the expression of proinflammatory cytokines through NF-*κ*B activation^33^. TLR4 expression is markedly elevated in DSS-induced colitis model mice^32,47,48^. TLR4 deficiency or targeted inhibition of the TLR4/NF-κB signaling pathway ameliorates the development of DSS-induced experimental colitis^31,49^. Interestingly, a previous study reported that the levels of PPARγ are decreased in both patients with active UC and CD patients^36^, while TLR4 expression was significantly increased in IBD patients, specifically in patients with active UC^35,36,50^. PPARγ plays an important role in suppressing the TLR4-mediated inflammatory response by decreasing LPS-induced NF-*κ*B activation in macrophages^37^. Thus, PPARγ is a negative regulator of the expression of proinflammatory cytokines^38^. Furthermore, it has been reported that PPARγ expression is negatively regulated by TDAG51, thereby promoting the expression of proinflammatory cytokines in macrophages^25^. We have also recently reported that TDAG51 acts as a corepressor of the transcriptional regulation of PPARγ by inhibiting transcriptionally active heterodimer formation between PPARγ and its obligatory dimer partner retinoid X receptor α^34^. Thus, we hypothesize that elevation of TDAG51 negatively regulates PPARγ activity or PPARγ expression per se in DSS-induced colitis model mice, thereby possibly promoting the production of inflammatory mediators via the TLR4/NF-κB signaling pathway. However, further intensive studies are needed to reveal the molecular mechanisms underlying the direct link between TDAG51 and PPARγ in the regulation of inflammatory mediator production in DSS-induced colitis model mice.

In conclusion, we report for the first time that TDAG51 deficiency plays a protective role against DSS-induced experimental colitis in mice. TDAG51 deficiency effectively prevents the development and progression of DSS-induced experimental colitis in mice by decreasing the production of inflammatory mediators. These findings suggest that TDAG51 is a novel regulator of the development of DSS-induced colitis and is a potential therapeutic target for IBD.

## Methods

### Mice

C57BL6/J mice were obtained from the Laboratory Animal Resource Center of the Korea Research Institute of Bioscience and Biotechnology (Ochang, Korea). TDAG51^−/−^ mice were generated on the C57BL/6J background as described previously^51^. The mice were housed 5 per cage in a room maintained at 22 ± 3 °C on a 12 h light–dark cycle (lights on at 8:00 a.m.) and provided ad libitum access to food and water. All tests were performed using 8-week-old male mice in the specific pathogen-free facility at Chungnam National University (Daejeon, Korea). All animal experiments were conducted in accordance with the ARRIVE guidelines and approved by the Animal Experiment Ethics Committee at Chungnam National University (approval Nos. CNU-01137, CNU-00674 and CNU-01097). All procedures were performed in accordance with relevant guidelines and regulations.

### DSS-induced colitis in mice

Acute colitis was induced in mice using DSS as previously described^27,29^. Briefly, 2.5% DSS (molecular weight 36,000–50,000; MP Bio Medicals, OH, USA) was orally administered in drinking water for 7 days, and then water was given for 5 days. The percentage body weight change and severity score were monitored daily during the experimental period as described previously^52,53^. The severity index was calculated as the sum of the severity scores for body weight loss (0: < 1%, 1: 1–5%, 2: 6–10% and 3: > 10%), stool consistency (0: normal pellets, 1: slightly loose feces, 2: loose feces and 3: watery diarrhea) and rectal bleeding (0: no rectal bleeding, 1: hemoccult-positive, 2: bloody and 3: heavy bleeding). The mice were sacrificed on day 5 for internal macroscopic examination and histopathological analysis. The spleen coefficient and colon length were carefully measured as previously described^27,54^. Feces in colons were removed by flushing with ice-cold PBS, and the colons were stored for western blotting, real-time PCR and histopathological analysis. Chronic colitis was induced in mice by 3 cycles of 1.7% DSS administration in drinking water for 4 days followed by water for 3 days as previously described^27,29,53^. The percentage body weight change and severity score were monitored daily during the experimental period. The mice were sacrificed on day 21 for internal macroscopic examination, western blotting, real-time PCR and histopathological analysis.

### Histological analysis and immunohistochemistry

Histological analysis was performed as described previously^19,55,56^. Briefly, isolated distal anorectal colons (1 cm) were fixed in 10% formalin for 48 h prior to paraffin embedding. Tissue sections (0.4 μm) were mounted on glass slides and stained with H&E. Villus structure and epithelial damage in the stained colon tissues were examined using an inverted microscope (DM-IRB Leica Microsystems, Wetzlar, Germany). Histopathological scores (loss of mucosal architecture, wall thickness and inflammatory cell infiltration) from the same area of the distal anorectal region samples were evaluated as described previously^57^. To further analyze mucus secretion, the sectioned slides were stained with Alcian blue according to the manufacturer’s instructions (Sigma–Aldrich, MO, USA). Immunohistochemistry was performed as previously described^15,58^. Briefly, the sectioned slides were immersed in PBS for 10 min, boiled in antigen retrieval buffer (10 mM Tris–EDTA, pH 9.0) for 20 min and immersed again in PBS for 10 min. Finally, the slides were soaked in 3% H_2_O_2_ for 10 min and washed with distilled water. Then, the sections were blocked with 5% bovine serum albumin in PBS for 1 h, incubated with primary antibody against TDAG51 (Santa Cruz Biotechnology, CA, USA) overnight at 4 °C, and washed three times with 1% Triton X-100 in PBS for 3 min. The sections were incubated with HRP-conjugated goat anti-mouse secondary antibody (SC-2005, Santa Cruz Biotechnology), developed using 3,3′-diaminobenzidine (brown color) and visualized with nuclear hematoxylin counterstain (blue color) under an inverted microscope (DM-IRB Leica Microsystems). The brown-stained cells were measured by ImageJ software (National Institutes of Health, Bethesda, MD, USA).

### Western blot analysis

Western blot analysis of colon tissue was performed as previously described^19,34,56^. The distal colon was homogenized in 1 ml of lysis buffer (25 mM Tris–HCl (pH 7.5), 150 mM NaCl, 1 mM EDTA, 1 mM NaF, 1 mM sodium orthovanadate, 1 mM phenylmethylsulfonyl fluoride, 5% glycerol and 0.5% Triton X-100). The lysate was separated twice by centrifugation at 13,500×*g* for 15 min at 4 °C, and the supernatant was transferred to a new tube. The supernatants were immunoblotted with anti-TDAG51 (Santa Cruz Biotechnology), anti-cyclooxygenase-2 (anti-Cox-2, Santa Cruz Biotechnology), anti-inducible nitric oxide synthase (anti-iNOS, Santa Cruz Biotechnology) and anti-β-actin (Sigma–Aldrich) antibodies.

### RNA extraction and quantitative real-time PCR

Relative mRNA levels were analyzed as described previously^19,34,56^. Briefly, total RNA was obtained from colon tissue using TRIzol reagent (MRC, OH, USA) according to the manufacturer’s instructions. To remove DSS from the RNA samples, the RNA was purified using the lithium chloride purification method as described previously^59^. Reverse transcription was performed using 1 μg of purified total RNA with M-MLV reverse transcriptase (Solgent, Daejeon, Korea). The cDNA was then subjected to real-time PCR amplification with primers using IQ SYBR Green Supermix and a real-time PCR detection system (Bio-Rad, CA, USA). The sequences of the primers used for real-time PCR amplification were as follows: TDAG51 (sense), 5′-GCG AGT CAG CCT TCT CTG CGC GCT-3′; TDAG51 (antisense), 5′-GGT GAG GAT GCA GCA CTT TTT CTT-3′; iNOS (sense), 5′-CCT TGT TCA GCT ACG CCT TC-3′; iNOS (antisense), 5′-AAG GCC AAA CAC AGC ATA CC-3′; Cox-2 (sense), 5′-TGG GGT GAT GAG CAA CTA TT-3′; Cox-2 (antisense), 5′-GGG TGC CAG TGA TAG AGT GT-3′; IL-6 (sense), 5′-GAG GAT ACC ACT CCC AAC AGA CC-3′; IL-6 (antisense), 5′-AAG TGC ATC ATC GTT GTT CAT ACA-3′; IL-1β (sense), 5′-ATG GCA ACT GTT CCT GAA CTC AAC T-3′; IL-1β (antisense), 5′-AGT GAT ACT GCC TGC CTG AAG CTC T-3′; and TNF-α (sense), 5′-CAT CTT CTC AAA ATT CGA GTG ACA A-3′; TNF-α (antisense), 5′-TGG GAG TAG ACA AGG TAC AAC CC-3′; β-Actin (sense), 5′-ATG AAG ATC CTG ACC GAG CG-3′; and β-Actin (antisense), 5′-TAC TTG CGC TCA GGA GGA GC-3. β-Actin was used as an internal control for normalization. Relative mRNA levels were analyzed as described previously^19^.

### Enzyme-linked immunosorbent assay (ELISA)

The concentration of cytokines in the serum was measured as described previously^19,60^. Blood samples were collected by retro-orbital bleeding, and the serum was separated at 1000×*g* for 10 min at 4 °C following 30 min of incubation at room temperature. IL-6 (BD Bioscience, CA, USA), IL-1β (Thermo Scientific, IL, USA), TNF-α (BD Bioscience) and nitric oxide (NO) assay kits (BD Bioscience) were used according to the manufacturer’s instructions. Cytokine levels were measured at 450 nm using a microplate reader (model 680; Bio-Rad).

### Luciferase reporter assay

Luciferase reporter assays were performed as described previously^34^. Briefly, 293/TLR4-MD2-CD14 cells (2.5 × 10^5^ cells/ml, InvivoGen, San Diego, CA, USA) were cotransfected with a luciferase reporter plasmid (0.2 μg), pcDNA3.1/His/LacZ (0.1 μg, Invitrogen, Carlsbad, CA, USA) and various expression plasmids (0.25–1.0 μg) in triplicate using TurboFect reagent (Fermentas, Glen Burnie, MD, USA). The murine IL-6 promoter-luciferase reporter (IL-6-Luc (−1277/ + 1)) and the murine TNF-α promoter-luciferase reporter (TNF-α-Luc (−1167/ + 155)) were described previously^61,62^; the murine IL-1β promoter-luciferase reporter (IL-1β-Luc (−2045/ + 23)) was generated by PCR-based subcloning into the pGL3-basic vector (Promega, Madison, WI, USA). At 24 h posttransfection, the transfected cells were stimulated with LPS (1 μg/ml) for 24 h. The cells were subjected to a luciferase assay using a luciferase activity assay system (Promega, Madison, WI, USA). All values represent luciferase activities normalized to β-galactosidase activities (Applied Biosystems, Bedford, MA, USA).

### Generation of TDAG51-deificent CT26 cell line

CT26 cells, a murine colorectal carcinoma cell line, were obtained from Korean Cell Line Bank (Seoul, Korea). TDAG51-deificent CT26 cell line was generated by introducing lentiviral CRISPR‒Cas9 system as described previously^63^. Briefly, CT26 cells were infected with CRISPR‒Cas9 lentivirus harboring the guide RNA sequences (5′-gcc gaa ccg tcc caa cct ag-3′) targeting the PHL domain of the murine TDAG51 with polybrene (8 μg/ml) for 6 h. The transduced cells were selected with puromycin (4 μg/ml) for 30 days to generate a stable cell line. To prepare a single cell clone, the puromycin-resistant cells were isolated in 96-well plates via serial dilution. Finally, TDAG51-deficient CT26 clones were analyzed by immunoblotting with anti-TDAG51 (Santa Cruz Biotechnology).

### Statistical analysis

All data are presented as the mean ± SD. Statistical analysis was performed by two-tailed Student's t test or one- or two-way ANOVA using Prism 9 Software (GraphPad software, San Diego, CA, USA). Statistical analysis for the real-time PCR and ELISA was performed using the nonparametric Mann–Whitney test. *P* < 0.05 was considered significant.

## Supplementary Information

Supplementary Figures.
