# Supplementary material for: TDAG51 deficiency attenuates dextran sulfate sodium-induced colitis in mice
Source: Sci Rep. 2022 Nov 30;12:20619. doi: 10.1038/s41598-022-24873-4 (PMC9712416; doi:10.1038/s41598-022-24873-4)
Supplement: Supplementary file 1 — Supplementary Figures. [file 41598_2022_24873_MOESM1_ESM.pptx]

## Slide 1
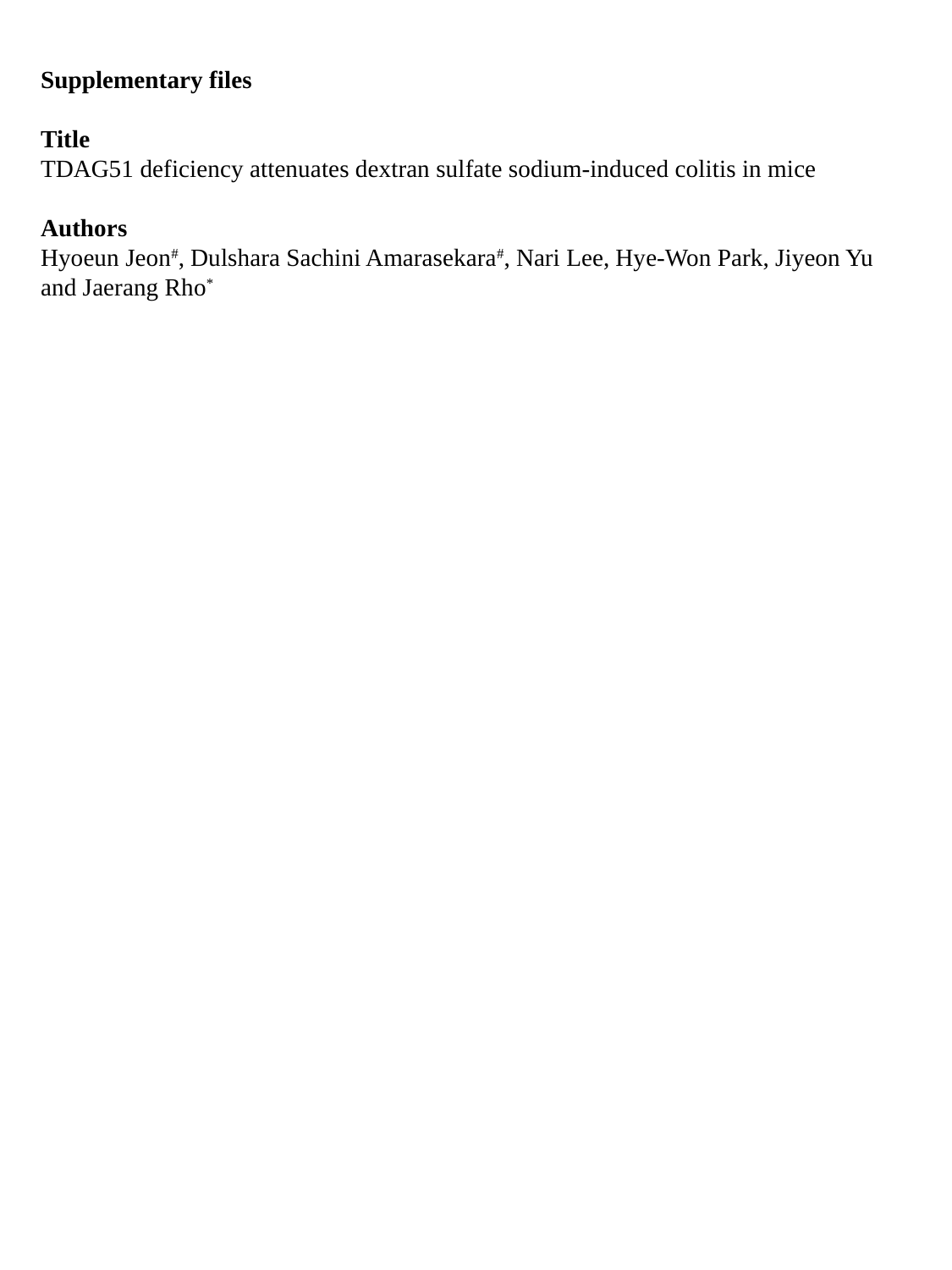

Supplementary files
Title
TDAG51 deficiency attenuates dextran sulfate sodium-induced colitis in mice
Authors
Hyoeun Jeon#, Dulshara Sachini Amarasekara#, Nari Lee, Hye-Won Park, Jiyeon Yu and Jaerang Rho*

## Slide 2
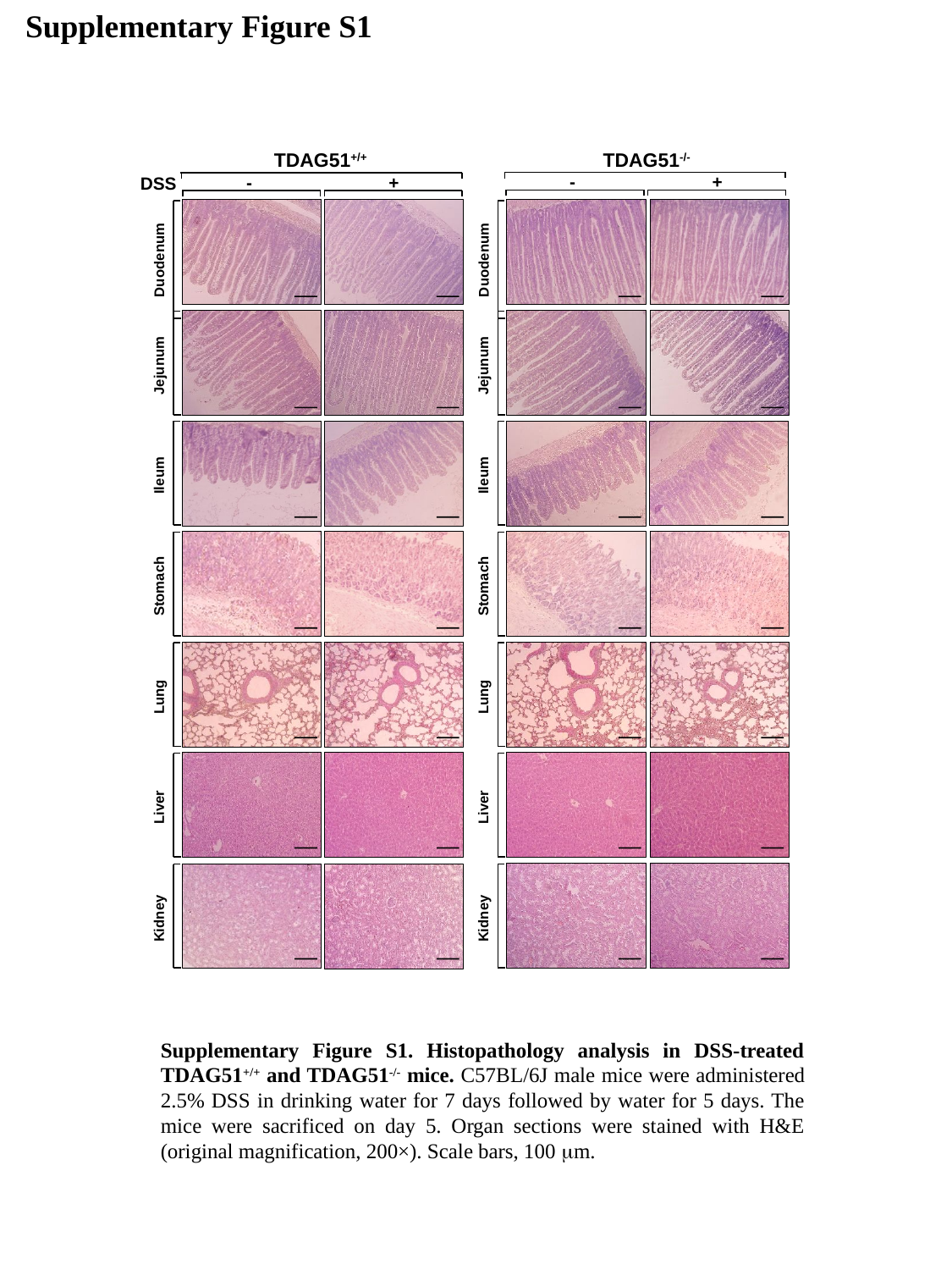

Supplementary Figure S1
TDAG51-/-
-
+
TDAG51+/+
-
+
DSS
Duodenum
Duodenum
Jejunum
Jejunum
Ileum
Ileum
Stomach
Stomach
Lung
Lung
Liver
Liver
Kidney
Kidney
Supplementary Figure S1. Histopathology analysis in DSS-treated TDAG51+/+ and TDAG51-/- mice. C57BL/6J male mice were administered 2.5% DSS in drinking water for 7 days followed by water for 5 days. The mice were sacrificed on day 5. Organ sections were stained with H&E (original magnification, 200×). Scale bars, 100 mm.

## Slide 3
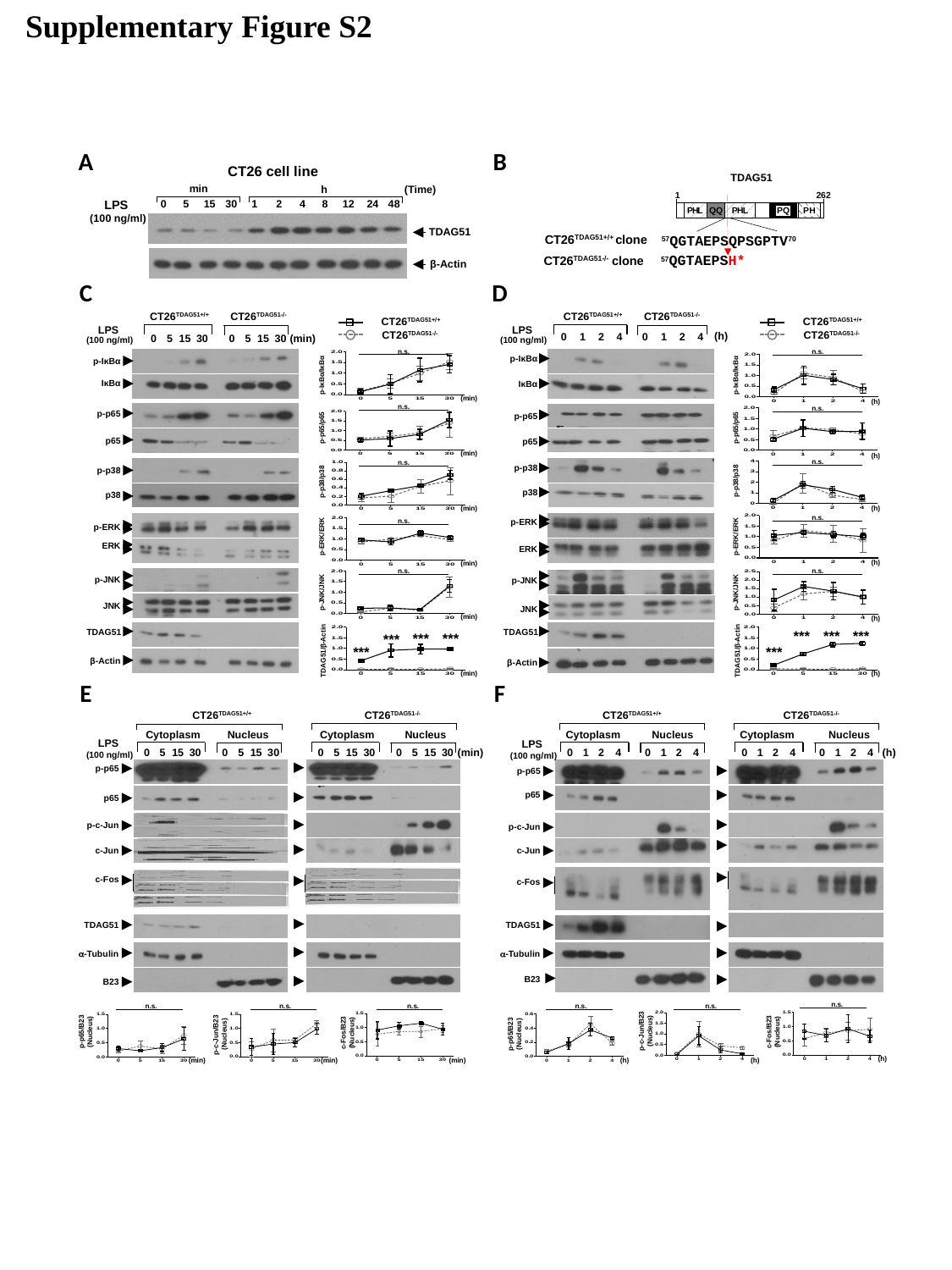

Supplementary Figure S2
A
B
CT26 cell line
TDAG51
262
1
QQ
PH
PHL
PHL
PQ
min
(Time)
h
LPS
(100 ng/ml)
0
5
15
30
1
2
4
8
12
24
48
 TDAG51
57QGTAEPSQPSGPTV70
CT26TDAG51+/+ clone
57QGTAEPSH*
CT26TDAG51-/- clone
β-Actin
C
D
CT26TDAG51+/+
CT26TDAG51+/+
CT26TDAG51-/-
CT26TDAG51-/-
CT26TDAG51+/+
CT26TDAG51-/-
CT26TDAG51+/+
CT26TDAG51-/-
LPS
(100 ng/ml)
LPS
(100 ng/ml)
(h)
0
1
2
4
0
1
2
4
0
5
15
30
0
5
15
30
(min)
n.s.
n.s.
p-IκBα
p-IκBα
p-IκBα/IκBα
p-IκBα/IκBα
IκBα
IκBα
(min)
(h)
n.s.
n.s.
p-p65
p-p65
p-p65/p65
p-p65/p65
p65
p65
(min)
(h)
n.s.
n.s.
p-p38
p-p38
p-p38/p38
p-p38/p38
p38
p38
(min)
(h)
n.s.
n.s.
p-ERK
p-ERK
p-ERK/ERK
p-ERK/ERK
ERK
ERK
(h)
(min)
n.s.
n.s.
p-JNK
p-JNK
p-JNK/JNK
p-JNK/JNK
JNK
JNK
(min)
(h)
TDAG51
TDAG51
***
***
***
***
***
***
TDAG51/β-Actin
TDAG51/β-Actin
***
***
β-Actin
β-Actin
(min)
(h)
E
F
CT26TDAG51+/+
CT26TDAG51-/-
CT26TDAG51+/+
CT26TDAG51-/-
Cytoplasm
Nucleus
Cytoplasm
Nucleus
Cytoplasm
Nucleus
Cytoplasm
Nucleus
LPS
(100 ng/ml)
LPS
(100 ng/ml)
0
5
15
30
0
5
15
30
0
5
15
30
0
5
15
30
0
1
2
4
0
1
2
4
0
1
2
4
0
1
2
4
(min)
(h)
p-p65
p-p65
p65
p65
p-c-Jun
p-c-Jun
c-Jun
c-Jun
c-Fos
c-Fos
TDAG51
TDAG51
a-Tubulin
a-Tubulin
B23
B23
n.s.
p-c-Jun/B23
(Nucleus)
(h)
n.s.
p-c-Jun/B23
(Nucleus)
(min)
n.s.
c-Fos/B23
(Nucleus)
(min)
n.s.
(min)
p-p65/B23
(Nucleus)
n.s.
c-Fos/B23
(Nucleus)
(h)
n.s.
p-p65/B23
(Nucleus)
(h)

## Slide 4
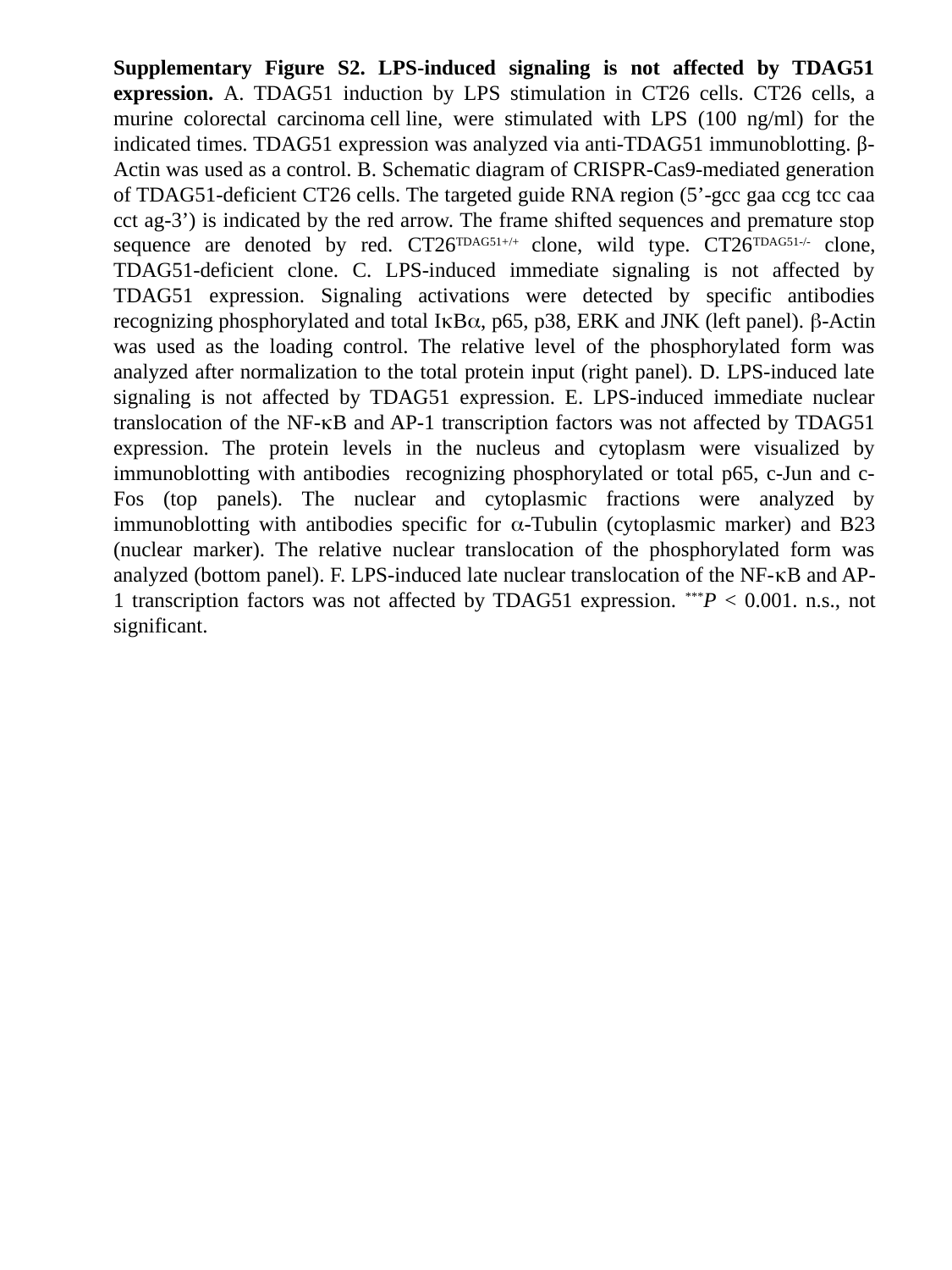

Supplementary Figure S2. LPS-induced signaling is not affected by TDAG51 expression. A. TDAG51 induction by LPS stimulation in CT26 cells. CT26 cells, a murine colorectal carcinoma cell line, were stimulated with LPS (100 ng/ml) for the indicated times. TDAG51 expression was analyzed via anti-TDAG51 immunoblotting. β-Actin was used as a control. B. Schematic diagram of CRISPR-Cas9-mediated generation of TDAG51-deficient CT26 cells. The targeted guide RNA region (5’-gcc gaa ccg tcc caa cct ag-3’) is indicated by the red arrow. The frame shifted sequences and premature stop sequence are denoted by red. CT26TDAG51+/+ clone, wild type. CT26TDAG51-/- clone, TDAG51-deficient clone. C. LPS-induced immediate signaling is not affected by TDAG51 expression. Signaling activations were detected by specific antibodies recognizing phosphorylated and total IκBα, p65, p38, ERK and JNK (left panel). β-Actin was used as the loading control. The relative level of the phosphorylated form was analyzed after normalization to the total protein input (right panel). D. LPS-induced late signaling is not affected by TDAG51 expression. E. LPS-induced immediate nuclear translocation of the NF-kB and AP-1 transcription factors was not affected by TDAG51 expression. The protein levels in the nucleus and cytoplasm were visualized by immunoblotting with antibodies recognizing phosphorylated or total p65, c-Jun and c-Fos (top panels). The nuclear and cytoplasmic fractions were analyzed by immunoblotting with antibodies specific for α-Tubulin (cytoplasmic marker) and B23 (nuclear marker). The relative nuclear translocation of the phosphorylated form was analyzed (bottom panel). F. LPS-induced late nuclear translocation of the NF-kB and AP-1 transcription factors was not affected by TDAG51 expression. ***P < 0.001. n.s., not significant.

## Slide 5
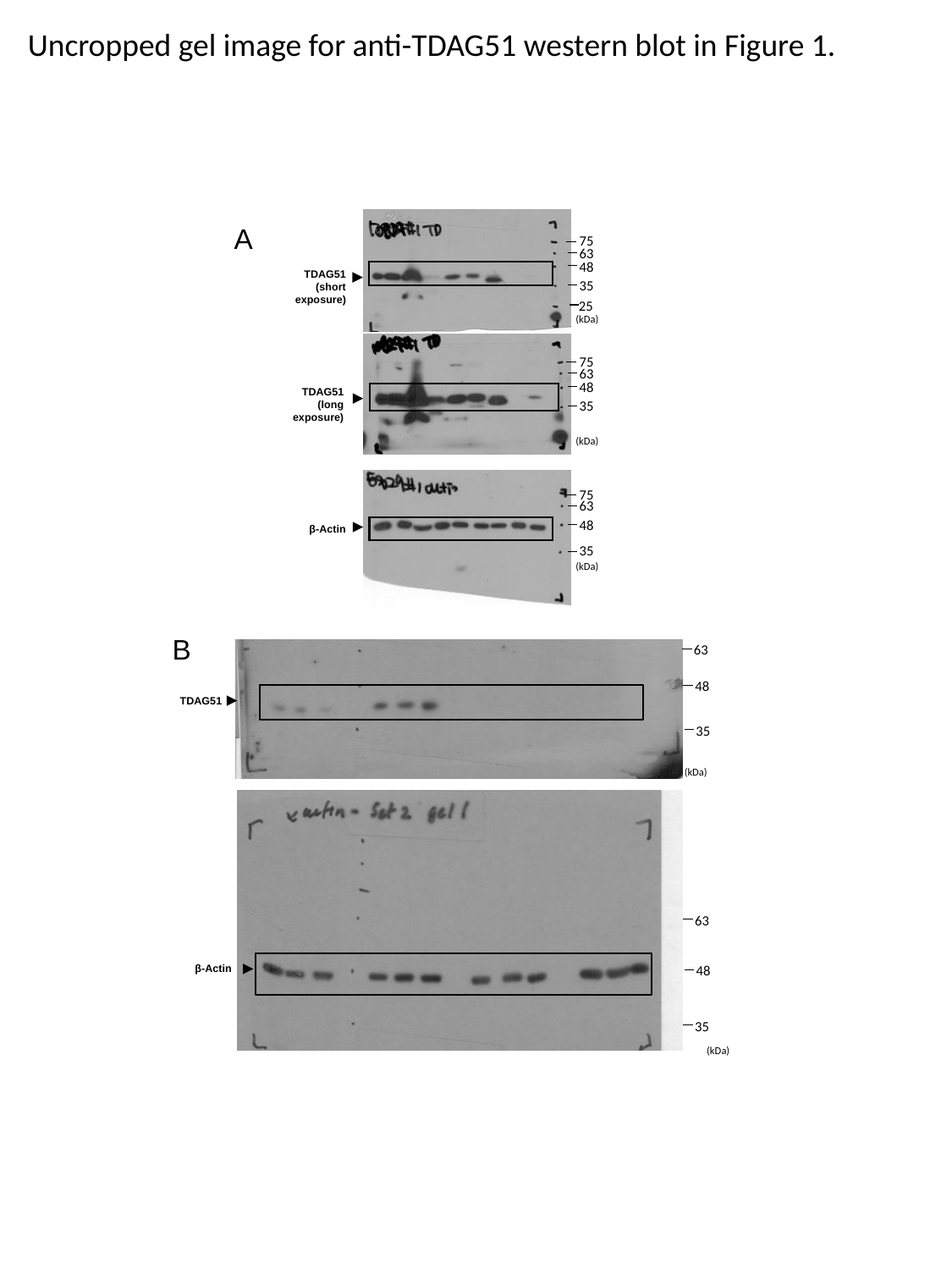

Uncropped gel image for anti-TDAG51 western blot in Figure 1.
A
75
63
48
TDAG51
(short exposure)
35
25
(kDa)
75
63
48
TDAG51
(long exposure)
35
(kDa)
75
63
48
β-Actin
35
(kDa)
B
63
48
TDAG51
35
(kDa)
63
β-Actin
48
35
(kDa)

## Slide 6
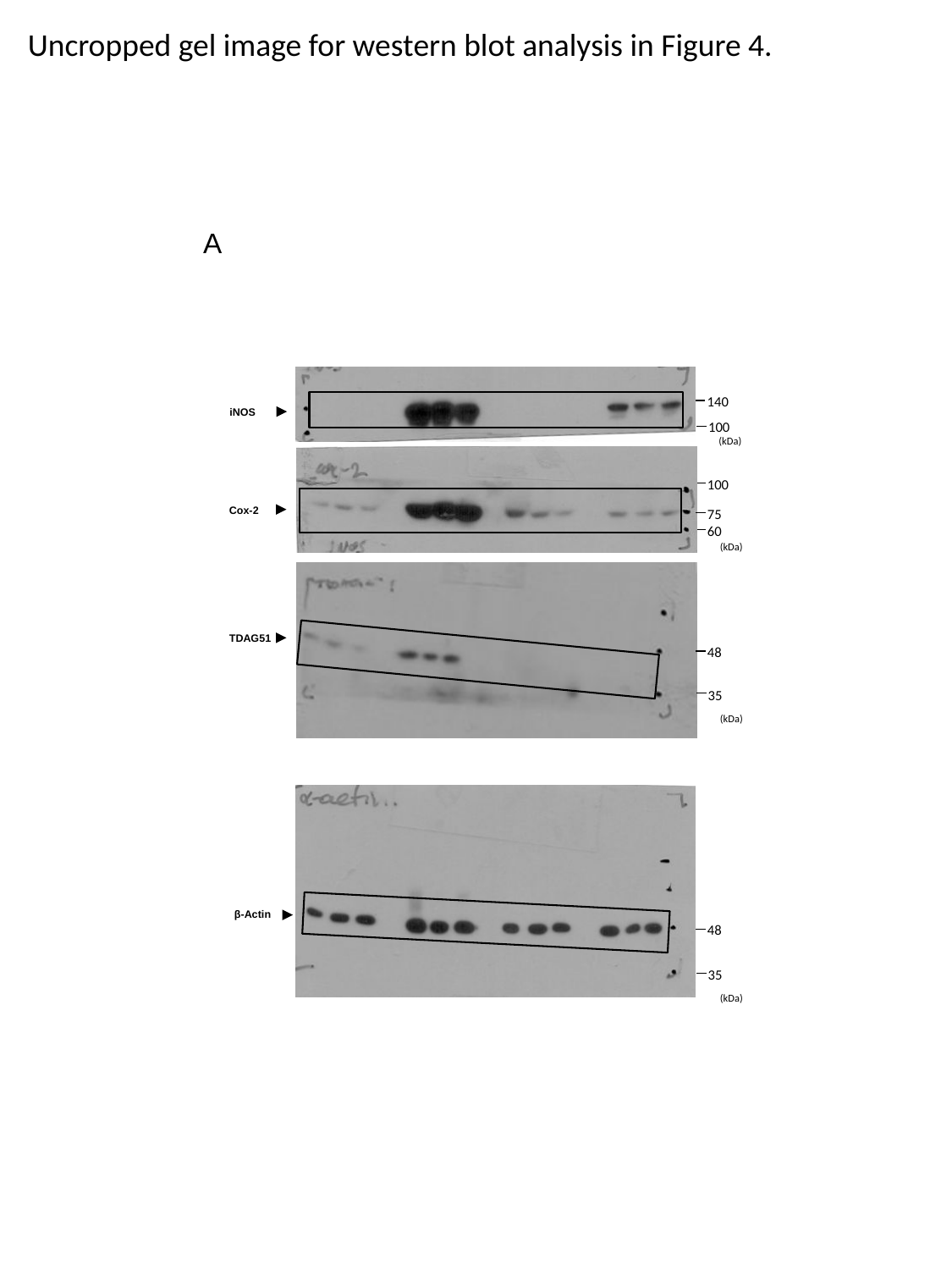

Uncropped gel image for western blot analysis in Figure 4.
A
140
iNOS
100
(kDa)
100
Cox-2
75
60
(kDa)
TDAG51
48
35
(kDa)
β-Actin
48
35
(kDa)

## Slide 7
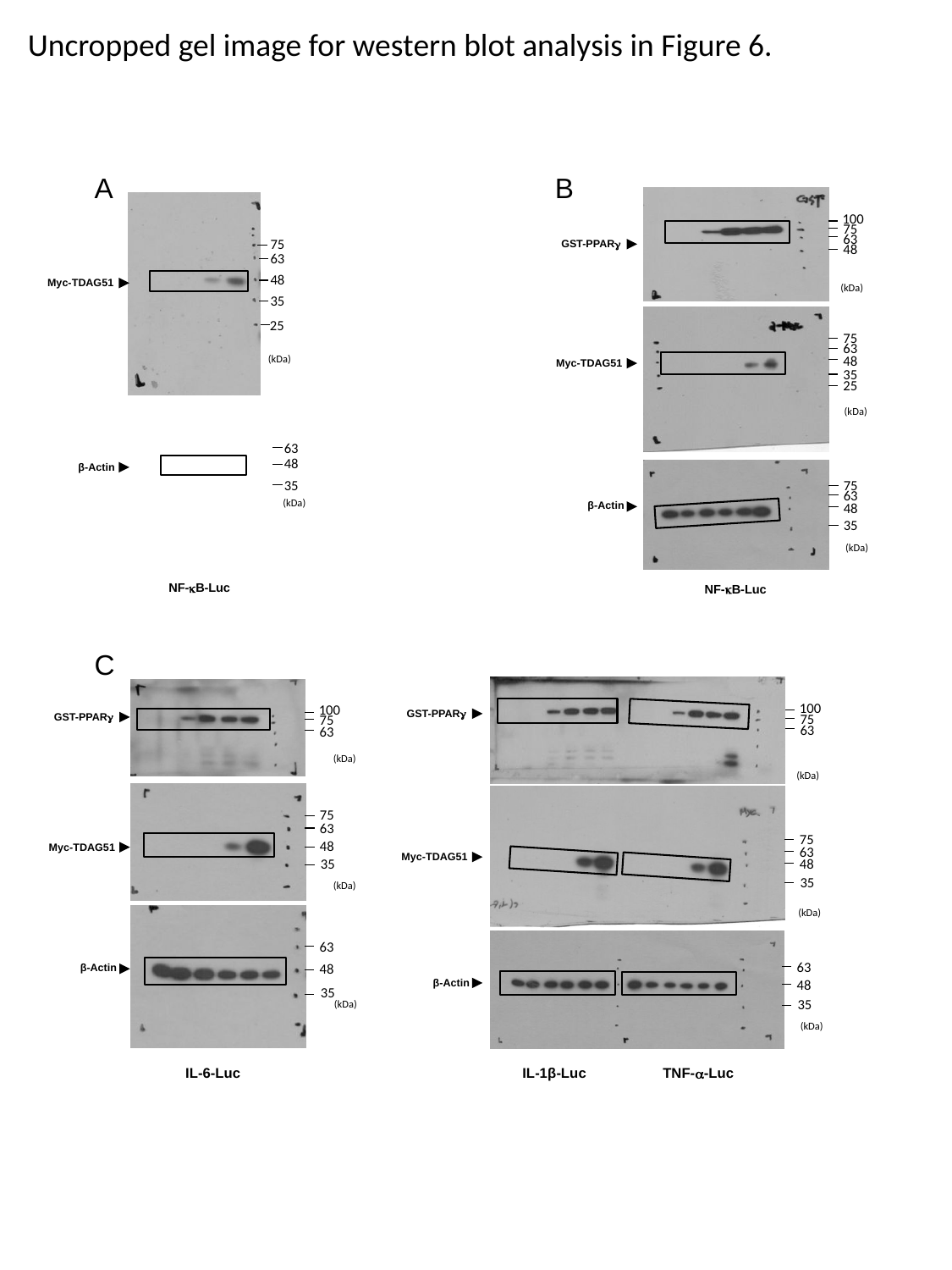

Uncropped gel image for western blot analysis in Figure 6.
A
B
100
75
63
75
GST-PPARg
48
63
48
Myc-TDAG51
(kDa)
35
25
75
63
(kDa)
48
Myc-TDAG51
35
25
(kDa)
63
48
β-Actin
75
35
63
(kDa)
β-Actin
48
35
(kDa)
NF-kB-Luc
NF-kB-Luc
C
100
100
GST-PPARg
75
GST-PPARg
75
63
63
(kDa)
(kDa)
75
63
75
48
Myc-TDAG51
63
Myc-TDAG51
48
35
35
(kDa)
(kDa)
63
63
48
β-Actin
48
β-Actin
35
35
(kDa)
(kDa)
IL-6-Luc
IL-1β-Luc
TNF-a-Luc

## Slide 8
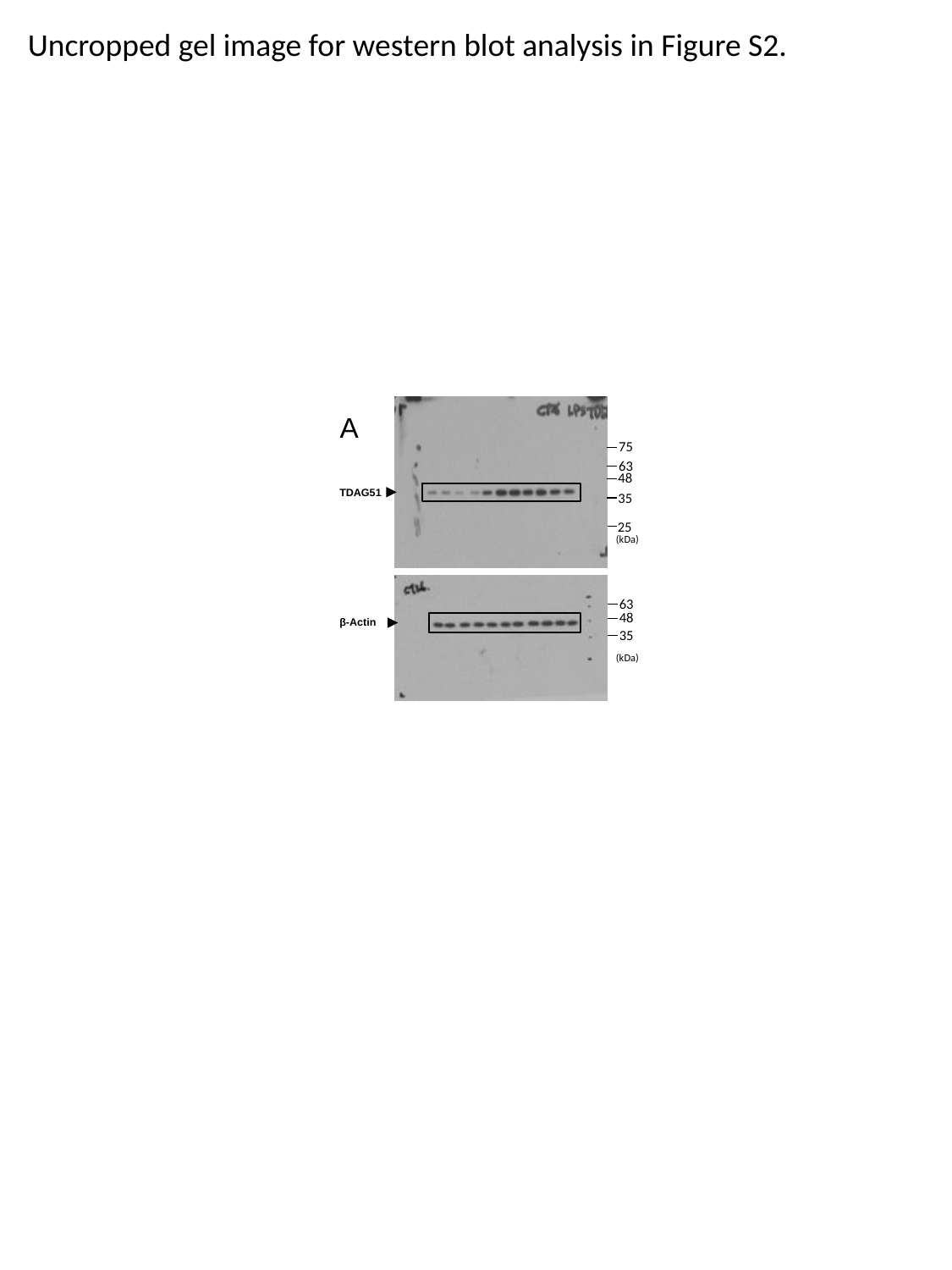

Uncropped gel image for western blot analysis in Figure S2.
A
75
63
48
TDAG51
35
25
(kDa)
63
48
β-Actin
35
(kDa)

## Slide 9
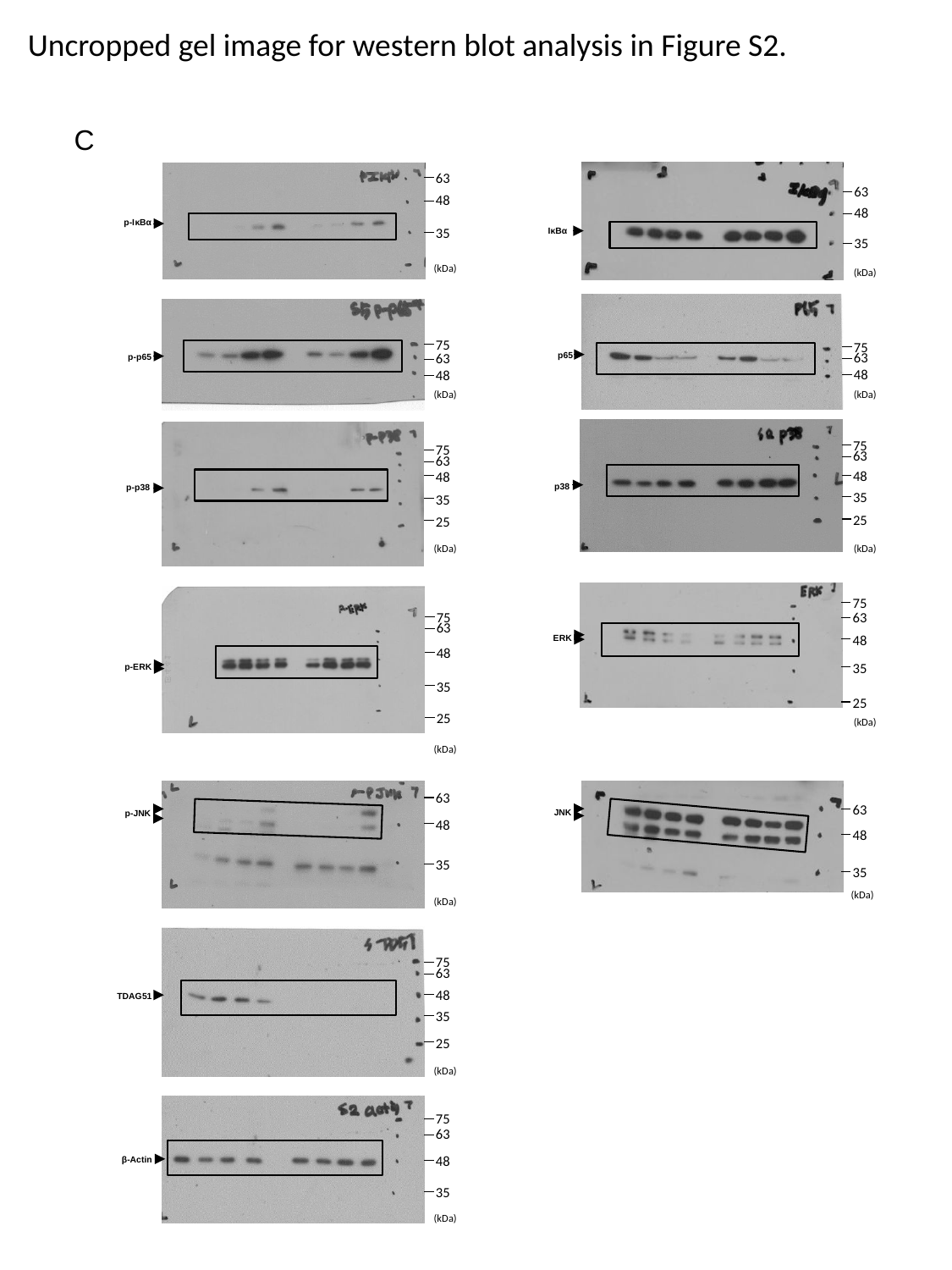

Uncropped gel image for western blot analysis in Figure S2.
C
63
63
48
48
p-IκBα
35
IκBα
35
(kDa)
(kDa)
75
75
63
63
p65
p-p65
48
48
(kDa)
(kDa)
75
75
63
63
48
48
p38
p-p38
35
35
25
25
(kDa)
(kDa)
75
63
75
63
48
ERK
48
35
p-ERK
35
25
25
(kDa)
(kDa)
63
63
JNK
p-JNK
48
48
35
35
(kDa)
(kDa)
75
63
48
TDAG51
35
25
(kDa)
75
63
48
β-Actin
35
(kDa)

## Slide 10
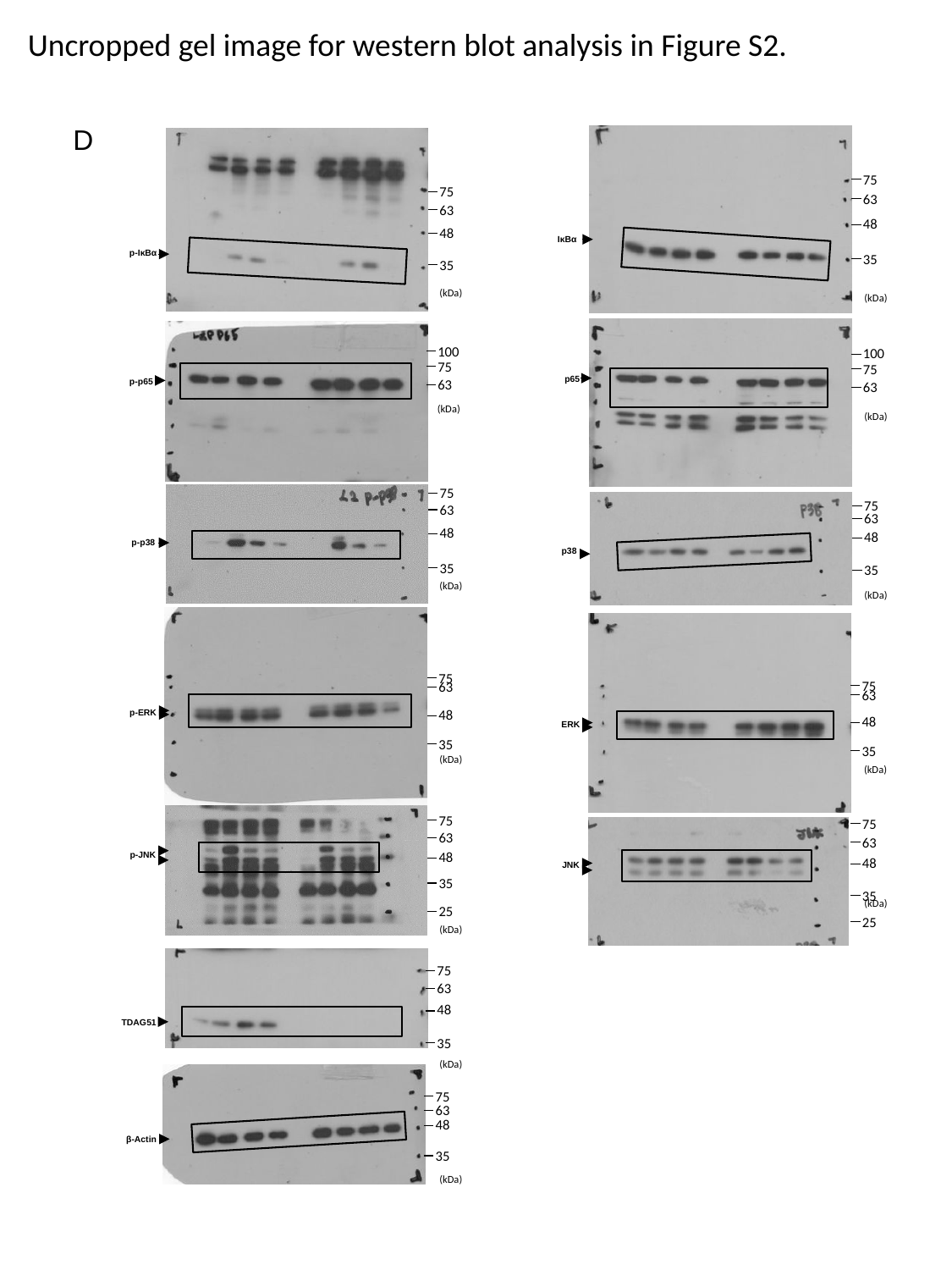

Uncropped gel image for western blot analysis in Figure S2.
D
75
75
63
63
48
48
IκBα
p-IκBα
35
35
(kDa)
(kDa)
100
100
75
75
p65
63
p-p65
63
(kDa)
(kDa)
75
75
63
63
48
48
p-p38
p38
35
35
(kDa)
(kDa)
75
75
63
63
48
p-ERK
48
ERK
35
35
(kDa)
(kDa)
75
75
63
63
48
p-JNK
48
JNK
35
35
(kDa)
25
25
(kDa)
75
63
48
TDAG51
35
(kDa)
75
63
48
β-Actin
35
(kDa)

## Slide 11
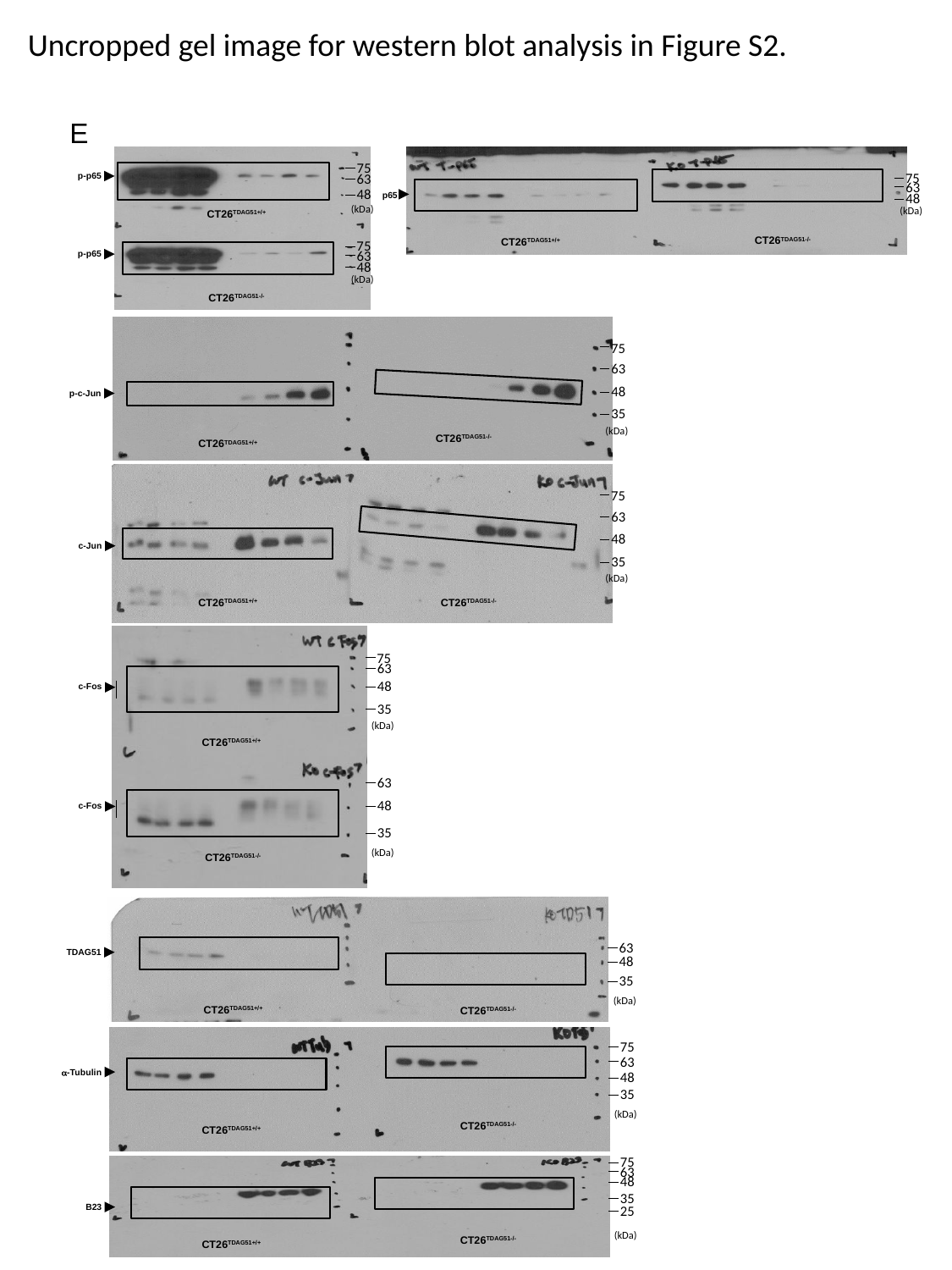

Uncropped gel image for western blot analysis in Figure S2.
E
75
75
p-p65
63
63
48
48
p65
(kDa)
(kDa)
CT26TDAG51+/+
CT26TDAG51-/-
CT26TDAG51+/+
75
63
p-p65
48
(kDa)
CT26TDAG51-/-
75
63
48
p-c-Jun
35
(kDa)
CT26TDAG51-/-
CT26TDAG51+/+
75
63
48
c-Jun
35
(kDa)
CT26TDAG51-/-
CT26TDAG51+/+
75
63
48
c-Fos
35
(kDa)
CT26TDAG51+/+
63
48
c-Fos
35
(kDa)
CT26TDAG51-/-
63
TDAG51
48
35
(kDa)
CT26TDAG51+/+
CT26TDAG51-/-
75
63
a-Tubulin
48
35
(kDa)
CT26TDAG51-/-
CT26TDAG51+/+
75
63
48
35
B23
25
(kDa)
CT26TDAG51-/-
CT26TDAG51+/+

## Slide 12
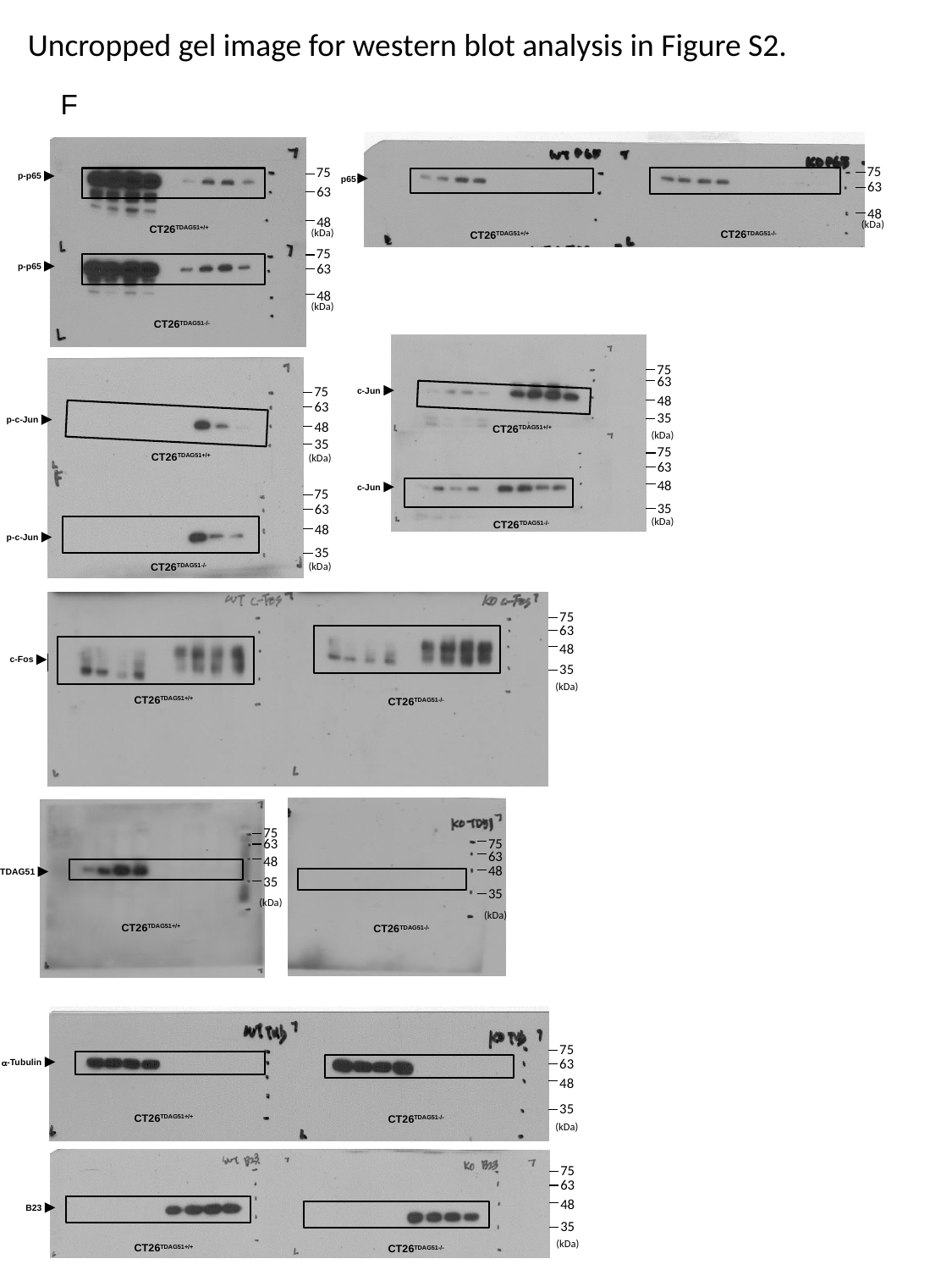

Uncropped gel image for western blot analysis in Figure S2.
F
75
75
p-p65
p65
63
63
48
48
(kDa)
CT26TDAG51+/+
(kDa)
CT26TDAG51-/-
CT26TDAG51+/+
75
63
p-p65
48
(kDa)
CT26TDAG51-/-
75
63
75
c-Jun
48
63
35
p-c-Jun
48
CT26TDAG51+/+
(kDa)
35
75
CT26TDAG51+/+
(kDa)
63
48
c-Jun
75
35
63
(kDa)
CT26TDAG51-/-
48
p-c-Jun
35
(kDa)
CT26TDAG51-/-
75
63
48
c-Fos
35
(kDa)
CT26TDAG51+/+
CT26TDAG51-/-
75
75
63
63
48
48
TDAG51
35
35
(kDa)
(kDa)
CT26TDAG51+/+
CT26TDAG51-/-
75
63
a-Tubulin
48
35
CT26TDAG51+/+
CT26TDAG51-/-
(kDa)
75
63
48
B23
35
(kDa)
CT26TDAG51+/+
CT26TDAG51-/-
